# Mechanism study of peptide GMBP1 and its receptor GRP78 in modulating gastric cancer MDR by iTRAQ-based proteomic analysis

**DOI:** 10.1186/s12885-015-1361-3

**Published:** 2015-05-06

**Authors:** Xiaojuan Wang, Yani Li, Guanghui Xu, Muhan Liu, Lin Xue, Lijuan Liu, Sijun Hu, Ying Zhang, Yongzhan Nie, Shuhui Liang, Biaoluo Wang, Jie Ding

**Affiliations:** State Key Laboratory of Cancer Biology and Xijing Hospital of Digestive Diseases, Xijing Hospital, Fourth Military Medical University, 127 Changle Western Road, Xi’an, 710032 China

**Keywords:** Gastric cancer, Multidrug resistance, Peptide GMBP1, GRP78

## Abstract

**Background:**

Multidrug resistance (MDR) is a major obstacle to the treatment of gastric cancer (GC). Using a phage display approach, we previously obtained the peptide GMBP1, which specifically binds to the surface of MDR gastric cancer cells and is subsequently internalized. Furthermore, GMBP1 was shown to have the potential to reverse the MDR phenotype of gastric cancer cells, and GRP78 was identified as the receptor for this peptide. The present study aimed to investigate the mechanism of peptide GMBP1 and its receptor GRP78 in modulating gastric cancer MDR.

**Methods:**

Fluorescence-activated cell sorting (FACS) and immunofluorescence staining were used to investigate the subcellular location and mechanism of GMBP1 internalization. iTRAQ was used to identify the MDR-associated downstream targets of GMBP1. Differentially expressed proteins were identified in GMBP1-treated compared to untreated SGC7901/ADR and SGC7901/VCR cells. GO and KEGG pathway analyses of the differentially expressed proteins revealed the interconnection of these proteins, the majority of which are involved in MDR. Two differentially expressed proteins were selected and validated by western blotting.

**Results:**

GMBP1 and its receptor GRP78 were found to be localized in the cytoplasm of GC cells, and GRP78 can mediate the internalization of GMBP1 into MDR cells through the transferrin-related pathway. In total, 3,752 and 3,749 proteins were affected in GMBP1-treated SGC7901/ADR and SGC7901/VCR cells, respectively, involving 38 and 79 KEGG pathways. Two differentially expressed proteins, CTBP2 and EIF4E, were selected and validated by western blotting.

**Conclusion:**

This study explored the role and downstream mechanism of GMBP1 in GC MDR, providing insight into the role of endoplasmic reticulum stress protein GRP78 in the MDR of cancer cells.

**Electronic supplementary material:**

The online version of this article (doi:10.1186/s12885-015-1361-3) contains supplementary material, which is available to authorized users.

## Background

Gastric cancer (GC) remains the fourth most common malignancy and the second leading cause of cancer-related death worldwide [1]. Although surgery is effective for most patients, chemotherapy remains the primary treatment for advanced gastric cancer [2]; nonetheless, therapies often fail due to the multidrug resistance (MDR) exhibited by some cancer cells. MDR is a phenomenon in which cancer cells that are exposed to one anti-cancer drug become resistant to several other chemotherapy drugs that are structurally and functionally different from the initial drug [3,4]. MDR is a multifactor event in which several mechanisms act simultaneously, including increased drug efflux, DNA repair activity, and altered survival and apoptotic signaling pathways [5-7]. Although there have been many pathogenesis studies on tumor MDR, the mechanisms of MDR are intricate and have not yet been fully elucidated [8]. Moreover, there is an urgent need to find novel approaches to reverse MDR in GC.

Short peptides with rapid blood clearance, high tissue penetration and diffusion, non-immunogenicity and a high affinity for target tumor cells have attracted great interest in recent years [9-11]. In a previous study using a phage display approach, we analyzed a peptide, GMBP1, that was specifically bound to the surface of MDR gastric cancer cells and that had the potential to be internalized into these cells and reverse the gastric MDR phenotype. GRP78 was later identified as a receptor for this peptide [12]. Importantly, exploring novel agents that can reverse MDR in GC is necessary for the improvement of chemotherapy in GC patients.

Proteomics is used as a powerful tool to accurately monitor and quantitatively detect changes in protein expression in response to drug treatment, and this approach has been widely used to investigate the mechanisms of action of chemicals on cancer cells [13-15]. Some technologies have been widely used in proteomics, including 2DE, SILAC, 2D-DIGE, and iTRAQ [16-20]. 2DE is an important proteomic technique and is widely used in comparative studies of protein expression levels. However, this technique has several disadvantages, including poor reproducibility between gels, low sensitivity in the detection of proteins in low concentrations and hydrophobic membrane proteins, limited sample capacity and a low linear range in visualization procedures [21,22]. Furthermore, only a limited number of proteins have been identified using the existing techniques. iTRAQ-based analysis, a technique that has been developed to quantitatively investigate changes in protein abundance in various biological samples with high accuracy and reproducibility [23,24], enables the differential labeling of peptides from distinct proteomes. In addition, the use of iTRAQ reagents with four to eight different tags allows for multiplexing ability [25]. High-throughout techniques can be used to screen MDR-related proteins and to study the mechanisms of gastric cancer drug resistance, and proteomics-based iTRAQ is an excellent choice for studying MDR mechanisms. Indeed, this approach has been successfully employed to identify differentially expressed proteins in gastric cancer [26].

Adriamycin and vincristine have been used to treat various cancers, and these drugs are accepted worldwide as first-line anti-cancer drugs for GC chemotherapy. However, their use remains limited because of the rapid development of MDR; thus, it is necessary to explore the mechanisms underlying this resistance. To further characterize the mechanisms of MDR, adriamycin-resistant SGC7901/ADR cells and vincristine-resistant SGC7901/VCR cells, which have been widely employed as cell culture models to investigate the mechanism underlying MDR in gastric cancer, were used in this study. These cell lines were derived from the human gastric cancer cell line SGC7901 by stepwise selection in vitro using adriamycin and vincristine and developed cross-resistance to other anticancer drugs, including cisplatin, adriamycin, etoposide, mitomycin C, and 5-fluorouracil (5-FU) [27]. Methods including FACS and immunofluorescence staining were used in this study to investigate the mechanism underlying the internalization of GMBP1. In addition, an iTRAQ-based proteomic approach coupled with bioinformatics, including GO and KEGG analyses, were also applied. Our work elucidates the molecular mechanism of GMBP1-induced reversal of MDR in GC, and the results presented here will undoubtedly provide important clues to the mechanisms of MDR in gastric cancer.

## Methods

### Cell lines and cell culture

Human MDR gastric adenocarcinoma adriamycin-resistant SGC7901/ADR and vincristine-resistant SGC7901/VCR cell lines were derived in our laboratory from the human gastric cancer cell line SGC7901 by stepwise selection in vitro using adriamycin and vincristine, respectively. The cells were cultured in RPMI-1640 medium (Thermo Scientific Hyclone, Beijing, China) containing 10% fetal bovine serum, 100 μg/ml streptomycin and 100 U/ml penicillin and incubated at 37°C with 5% CO_2_ in a humidified incubator. To maintain the MDR phenotype, vincristine (final concentration, 1 μg/ml) was added to the culture medium of the SGC7901/VCR cells, and adriamycin (final concentration, 0.5 μg/ml) was added to the culture medium of the SGC7901/ADR cells. Adriamycin (ADR) and vincristine (VCR) were dissolved in normal saline at the indicated concentrations.

### Transient transfection

For knockdown of GRP78, GC cells were transfected with a small interfering RNA (siRNA) targeting GRP78: sense 5′-GGAGCGCAUUGAUACUAGATT-3′ and antisense 5′-UCUAGUAUCAAUGCGCUCCTT-3′ [28]. siRNA targeting green fluorescent protein (GFP) was purchased from GenePharma (Shanghai, China) and served as a negative control. Both siRNAs were used at a final concentration of 80 nmol/l. The cells were transfected in six-well plates according to the manufacturer’s instructions. Ten microliters of each siRNA was used with 5 μl of Lipofectamine 2000 per well. The transfected cells were monitored for GFP under a fluorescence microscope.

### Immunofluorescence staining

Cells were cultured on cover slips and fixed with acetone at 4°C for 30 min, blocked with 10% normal rabbit serum, and incubated with a goat anti-human GRP78 antibody (1:500; Abcam, USA) overnight at 4°C. Subsequently, the cells were incubated with a secondary FITC-conjugated anti-goat antibody (1:1,000; Invitrogen, CA, USA) for 1 h at 37°C. A drop of Prolong Gold anti-fade reagent with DAPI (Invitrogen, CA, USA) was added before the cell images were acquired using a FLUOVIEW FV1000 laser scanning confocal microscope (Olympus, Tokyo, Japan). PBS and control siRNA were used as a negative control.

### Flow cytometric analysis for uptake assays

Cells were cultured in serum-free RPMI-1640 medium. After 24 h, the cells were trypsinized, centrifuged at 1,000 rpm for 5 min, harvested and washed with ice-cold PBS twice. The expression level of GMBP1-GRP78 was measured by staining the cells with FITC-conjugated GMBP1 in PBS containing 0.05% bovine serum albumin on ice. FITC-GMBP1 (200 μg/ml) was incubated with the cells in growth medium for 1 h at 37°C, and the cells were then washed twice with ice-cold PBS. After removing unbound FITC-GMBP1 by washing the cells extensively in PBS, the surface immunofluorescence of viable cells was measured using a flow cytometer. FITC-URP was used as a negative control.

### Double immunofluorescence staining

Cells were seeded on cover slips at a density of 10^6^ cells/ml; experiments were conducted at 24–72 h post-seeding. The multidrug-resistant gastric cells SGC7901/ADR and SGC7901/VCR with GMBP1 were doubly labeled as follows. Briefly, the cells were serum-starved for 2 h in RPMI-1640 medium. The cells were first incubated with FITC-GMBP1 in growth medium at 200 μg/ml for 1 h at 37°C in the dark and then washed twice with ice-cold PBS. The cells were then incubated with Alexa Fluor 594-transferrin (25 μg/ml) at 4°C for 3 h in the dark to stop receptor-mediated endocytosis [29]; the cells were then incubated at 37°C for 30 min to initiate the uptake of FITC-GMBP1, after which the cells were washed twice with ice-cold PBS. The cell nuclei were stained using 4, 6-diamidino-2-phenylindole (DAPI). Cell images were acquired using a FLUOVIEW FV1000 laser scanning confocal microscope (Olympus, Tokyo, Japan).

### Protein sample preparation and iTRAQ labeling

The treated and untreated SGC7901/ADR and SGC7901/VCR cells were harvested and lysed in lysis buffer and centrifuged at 15,000 rpm for 30 min at 4°C. The supernatants were collected, and the total protein concentration was determined using a Bradford protein assay kit. For each sample, 100 μg of protein was precipitated by adding six volumes of cold acetone and incubating at −20°C for 4 h. The precipitated protein was dissolved in solution buffer and denatured, and the cysteines were then blocked according to the manufacturer’s instructions (Applied Biosystems). Each sample was digested with 20 μl of 0.25 μg/μl trypsin (Promega) solution at 37°C overnight. iTRAQ labels 113 and 118 were used to separately label the control samples SGC7901/ADR and SGC7901/VCR, respectively, and the labels 115 and 119 were used to label the corresponding GMBP1-treated samples. The labeled samples were pooled before further analysis.

### Strong cation exchange chromatography separation

To reduce sample complexity during the LC-MS/MS analysis, the pooled samples were diluted 10-fold with HPRP buffer A (10 mM KH_2_PO_4_ in 25% acetonitrile at pH 3.0) and separated using a 2.1 × 200 mm polysulfoethyl A HPRP column (Poly LC, Columbia, MD, USA). The column was eluted with a gradient of 0–25% HPRP buffer B (10 Mm KH_2_PO_4_ at pH 3.0 in 25% acetonitrile containing 350 mM KCl) over 30 min followed by a gradient of 25-100% HPRP buffer B over 40 min. The fractions were collected at 1-min intervals. These HPRP fractions were lyophilized in a vacuum concentrator and subjected to C18 clean-up using a C18 Discovery DSC-18 SPE column (Thermo). The cleaned fractions were then lyophilized again and stored at −20°C until analyzed by mass spectrometry.

### Nano-LC-MS/MS analysis

The mass spectrometric analysis was performed using a nano-LC column coupled online to a QStarXL mass spectrometer (Applied Biosystems). Peptides were loaded onto a 75 cm × 10 cm, 3-mm fused silica C18 capillary column, and mobile phase elution was performed using buffer A (0.1% formic acid in 2% acetonitrile/98% Milli-Q water) and buffer B (0.1% formic acid in 98% acetonitrile/2% Milli-Q water). The peptides were eluted using a gradient from 2% buffer B to 100% buffer B over 90 min at a flow rate of 300 nl/min. The LC eluent was directed to an ESI source for Q-TOF-MS analysis. The mass spectrometer was set to perform information-dependent acquisition (IDA) in the positive ion mode for a selected mass range of 300–2,000 m/z. Peptides with +2 to +4 charge states were selected for tandem mass spectrometry, and the time of summation of MS/MS events was set to 3 s. The two most abundantly charged peptides above a 10-count threshold were selected for MS/MS and were dynamically excluded for 60 s with a ±50-mmu mass tolerance.

### Protein identification and relative quantization

The raw data were analyzed using Proteome Discoverer 1.4 (Thermo Fisher Scientific). The software was connected to a Mascot Search Engine server version 2.2.4 (Matrix Science, London, UK) and to a Sequest Search Engine version 28.0 (Thermo Fisher Scientific). The confidence value for each peptide was calculated based on the agreement between the experimental and theoretical fragmentation patterns. Each protein was assigned a confidence score (0% to 100%) based on the confidence scores of its constituent peptides based on unique spectral patterns. Proteins with confidence scores of greater than 90% and with at least 1 peptide of 95% identification confidence were used for further quality control and differential expression analyses. Each protein also received quantitative scores for each of the eight-iTRAQ tags to calculate the relative expression levels. In this experiment, the relative expression levels of proteins in different samples were calculated using a normal sample as the reference sample.

### Bioinformatic analysis of differentially expressed proteins

The theoretical pI values and molecular weights (MWs) of the identified proteins were obtained from the UniProt protein sequence database. Functional enrichment analysis was performed using Gene Ontology (GO) (http://www.geneontology.org/), and GO annotation was applied to describe the functions of the differentially expressed proteins, which were classified into three major categories: cellular component, molecular function, and biological process [30]. Pathway analysis was performed by KEGG mapping. Both assays proved statistically significant with p-values of less than 0.01 and 0.05, respectively.

### Western blotting assay

Proteins were extracted from cells in log-phase growth and were separated using SDS–PAGE. A western blot analysis was then performed according to standard procedures. Briefly, total proteins were resolved by 10% SDS-PAGE and then transferred to nitrocellulose membranes. After incubating with primary antibodies at 4°C overnight, the nitrocellulose membranes were then washed three times with Tris-buffered saline containing Tween-20 (TBST) and incubated with horseradish peroxidase-conjugated secondary antibodies (1:2,000; Santa Cruz, USA) for 2 h at room temperature. The membranes were then washed again in TBS-T and visualized using an Enhanced ChemiLuminescence Kit (ECL-Kit, Santa Cruz, USA). Anti-CTBP2 and anti-EIF4E primary antibodies used for western blotting (1:500 dilutions; Abcam, USA), and an anti-β-actin antibody (Beyotime, China) was used as an internal reference. The experiments were repeated three times.

### Statistical analysis

GraphPad Prism and image J software were used for data analysis. The results are presented as the mean ± standard deviation. Student’s *t*-test was performed to evaluate differences between the western blotting analysis results. P-values of less than 0.05 were considered statistically significant.

## Results

### Subcellular localization of GMBP1 and its receptor GRP78 in multidrug-resistant gastric cells

**Figure 1:**
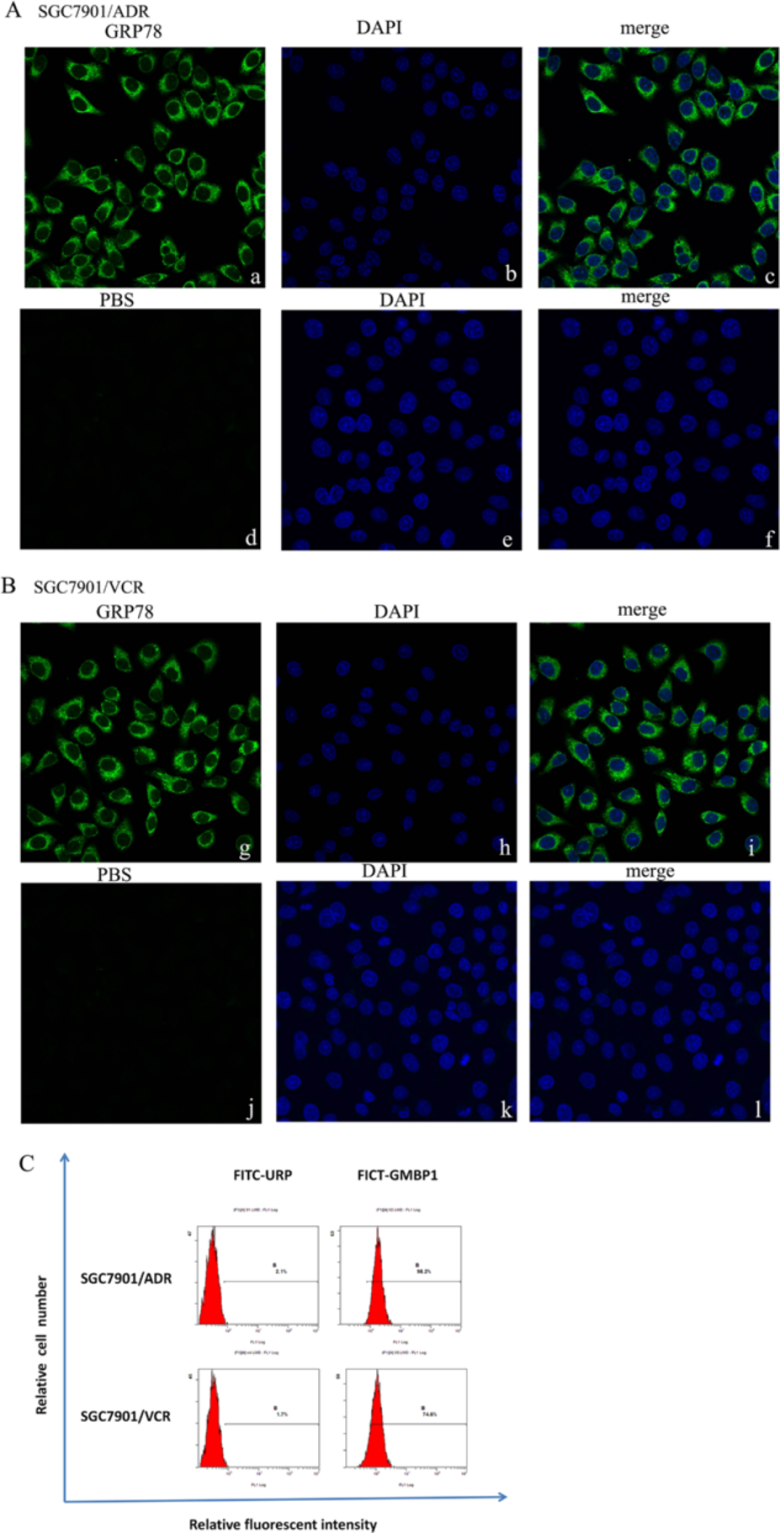
Subcellular localization of GMBP1 and its receptor GRP78 in SGC7901/ADR and SGC7901/VCR. **(A-B)**: a, d, g, j: The cytoplasmic localization of internalized GRP78 (green). b, e, h, k: Nuclear staining with 4, 6-diamidino-2-phenylindole (DAPI; blue). c, f, i, l: Merged images showing the relationship between GRP78 and the nucleus. **(C)**: The internalization of the GMBP1 peptide into SGC7901/ADR and SGC7901/VCR cells. FITC-GMBP1 bound to SGC7901/ADR and SGC7901/VCR cells exhibited higher fluorescence intensity than the negative control FITC-URP group.

In the present study, the localization of GMBP1 in multi-drug resistant gastric cells SGC7901/ADR and SGC7901/VCR was demonstrated by immunofluorescence staining and flow cytometric assays. As shown in the immunofluorescence staining assay, in both cell lines, positive staining was mainly located in the cytoplasm and was observed as a green color in the FITC-GMBP1 group; in contrast, the PBS group exhibited no staining (Figure 1(A,B)). Similarly, flow cytometry analysis showed higher fluorescence intensity for FITC-GMBP1 bound to SGC7901/ADR and SGC7901/VCR cells compared to the negative control FITC-URP group (Figure 1(C)). These results demonstrate that GMBP1 and its receptor GRP78 were located in the cytoplasm of gastric cancer cells but not in the control group.

### Internalization of the GMBP1 peptide into multidrug-resistant gastric cells

**Figure 2:**
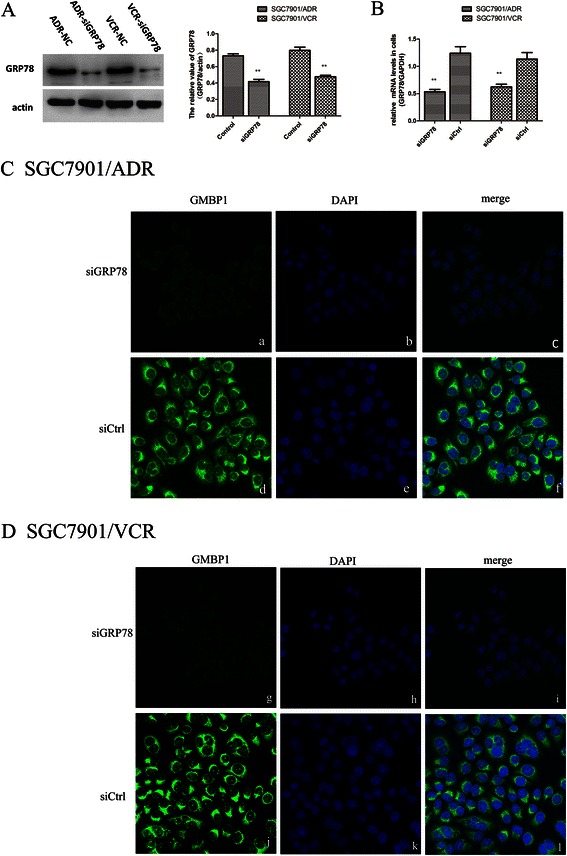
Internalization of the GMBP1 peptide into SGC7901/ADR and SGC7901/VCR cells. **(A)**: Relative expression of GRP78 in SGC7901/ADR cells and SGC7901/VCR cells transfected with control-siRNA or GRP78-siRNA, which were confirmed western blot analysis. The values represent the means from three separate experiments, and the error bars represent the SEM (*P < 0.01). **(B)**: The relative mRNA level of GRP78 in SGC7901/ADR and SGC7901/VCR cells. **(C, D)**: a, d, g, j: The cytoplasmic localization of FITC-GMBP1 (green). b, e, h, k: Nuclear staining with 4, 6-diamidino-2-phenylindole (DAPI; blue). c, f, i, l: Merged images showing the relationship between GMBP1 and the nucleus.

To explore the role of GRP78 in the internalization of the GMBP1 peptide into multi-drug resistant gastric cells, the specific downregulator GRP78 siRNA (siGRP78) and control siRNA (siCtrl) were transfected into SGC7901/ADR and SGC7901/VCR cells. Western blot and RT-PCR analyses showed that the transfection of SGC7901/ADR and SGC7901/VCR cells with the specific GRP78 siRNA resulted in a marked inhibition of GRP78 protein expression and decreased mRNA levels compared to cells transfected with the control siRNA (p < 0.01) (Figure 2(A,B)). An immunofluorescence staining assay showed that the control group incubated with FITC-GMBP1 did exhibit green staining (Figure 2(C, D)); the same results (data not shown) were observed using the GRP78 inhibitor. These results suggest that GMBP1 was internalized into the multi-drug resistant gastric cells and that this internalization was receptor mediated.

### The mechanism of GRP78-mediated GMBP1 internalization in multidrug-resistant gastric cells

**Figure 3:**
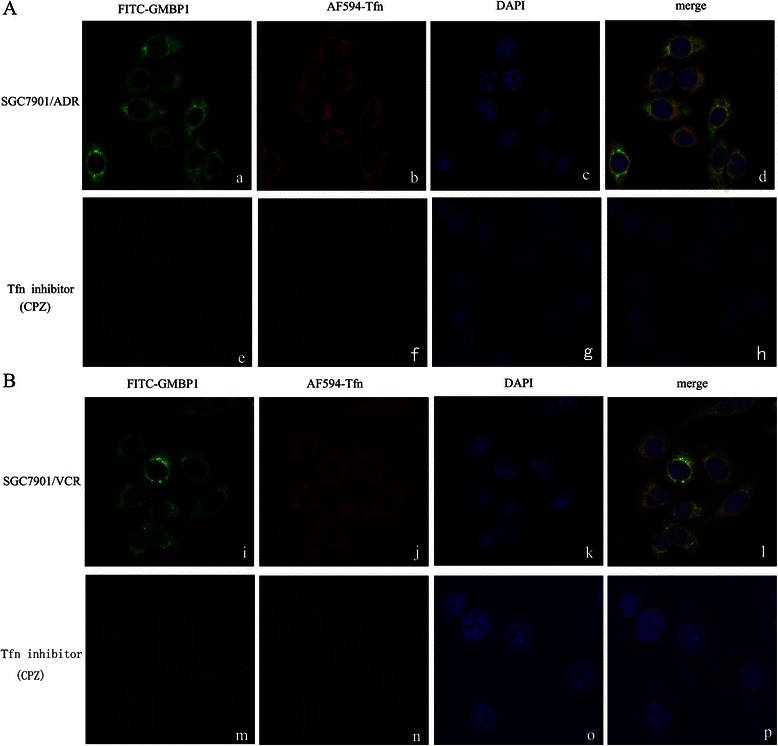
The mechanism of GRP78-mediated GMBP1 internalization into SGC7901/ADR and SGC7901/VCR cells. **(A, B)**: a, i: FITC-GMBP1 observed on the cell surface and in the cytoplasm. b, j: Alexa Fluor 594-transferrin observed primarily in the cytoplasm. e, m: Internalization of FITC-GMBP1 was strongly decreased after blocking the uptake of Alexa Fluor 594-transferrin. f, n: Chlorpromazine largely blocked the uptake of Alexa Fluor 594-transferrin. c, g, k, o: Nuclear staining with 4, 6-diamidino-2-phenylindole (DAPI; blue). d, h, l, p: Merged images showing the relationship between GMBP1 and transferrin.

To further characterize the mechanism of GRP78-mediated GMBP1 internalization in multi-drug resistant gastric cells, a double immunofluorescence staining assay was used. The effects of GRP78-mediated GMBP1 internalization on the uptake of Alexa Fluor 594-transferrin by the multi-drug resistant gastric cells are shown in Figure 3. Cells were doubly labeled with FITC-GMBP1 (green) and Alexa Fluor 594-transferrin (red) under control conditions at 37°C for 30 min; both FITC-GMBP1 and Alexa Fluor 594-transferrin were internalized, and FITC-GMBP1 was observed on the cell surface and in the cytoplasm (Figure 3(a, i)), whereas transferrin was observed primarily in the cytoplasm (Figure 3(b, j)). The labeled proteins were found to colocalize in the cytoplasm and perinuclear regions of the cells (Figure 3(d, l)). Furthermore, when chlorpromazine (CPZ), an inhibitor of clathrin-dependent endocytosis [31,32], blocked transferrin uptake, the red fluorescence of Alexa Fluor 594-transferrin was barely detectable (Figure 3(f, n)), and the green fluorescence of FITC-GMBP1 was also greatly reduced (Figure 3(e, m)). These results showed that the GRP78-mediated internalization of GMBP1 occurred through a clathrin-independent, transferrin-related pathway.

### Proteome analysis

**Figure 4:**
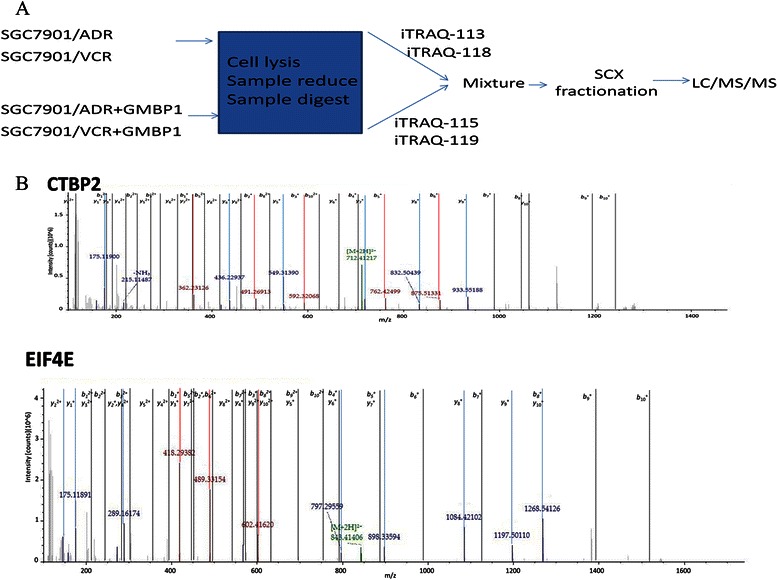
The flow chat of iTRAQ method and representative MS/MS spectrum of target proteins. **(A)**: A schematic flow of the iTRAQ method. **(B)**: A representative MS/MS spectrum showing CTBP2 and EIF4E peptides.

Our goal was to identify differentially expressed proteins that are related to MDR in GC and subsequently, to validate a subset of these proteins. We used cells from the multidrug-resistant gastric cell lines SGC7901/ADR and SGC7901/VCR for this study, and a schematic flow of the iTRAQ method used is shown in Figure 4. To increase the coverage of protein identification and/or the confidence in the data generated, proteins from these cell lines were labeled with iTRAQ reagents (the 113 tag for cell line SGC7901/ADR and the 115 tag for GMBP1-treated SGC7901/ADR cells). Thus, the ratio of labels 115 and 113 would indicate the relative abundance of MDR-related proteins. Similarly, proteins from these cell lines were also labeled with iTRAQ reagents (the 118 tag for cell line SGC7901/VCR and the 119 tag for GMBP1-treated SGC7901/VCR cells). Again, the ratio of labels 119 and 118 would also indicate the relative abundance of MDR-related proteins. To examine the biological reproducibility of the results, duplicate protein samples were obtained from both control and GMBP1-treated groups in two independent experiments. The iTRAQ analysis was performed in double-duplex style.

All the unique proteins were identified (false discovery rate < 1%) in the two biological replicates, and linear regression analyses were performed to examine the biological reproducibility of the results. Although the relative quantification analysis conducted using Protein Pilot 3.0 software includes statistical analysis, most methods are prone to technical variation; therefore, we included an additional 1.5-fold change and a 0.8-fold change cutoff for all iTRAQ ratios to reduce false positives for the selection of differentially expressed proteins. In total, 143 proteins were differentially expressed in the GMBP1-treated SGC7901/ADR cells compared with the SGC7901/ADR cells: 95 proteins were upregulated and 48 were downregulated (Additional file 1). For the SGC7901/VCR cells, 217 proteins were expressed differently following GMBP1 treatment compared to the control cells: 129 were upregulated, and 88 were downregulated (Additional file 2). Protein properties, including pI, molecular weight (MW), and number of residues, were calculated using PEPSTATS in EMBOSS. The grand average hydropathy (GRAVY) values were calculated as the arithmetic mean of the sum of the hydropathic indices of each amino acid.

### Classification of differentially expressed proteins

**Figure 5:**
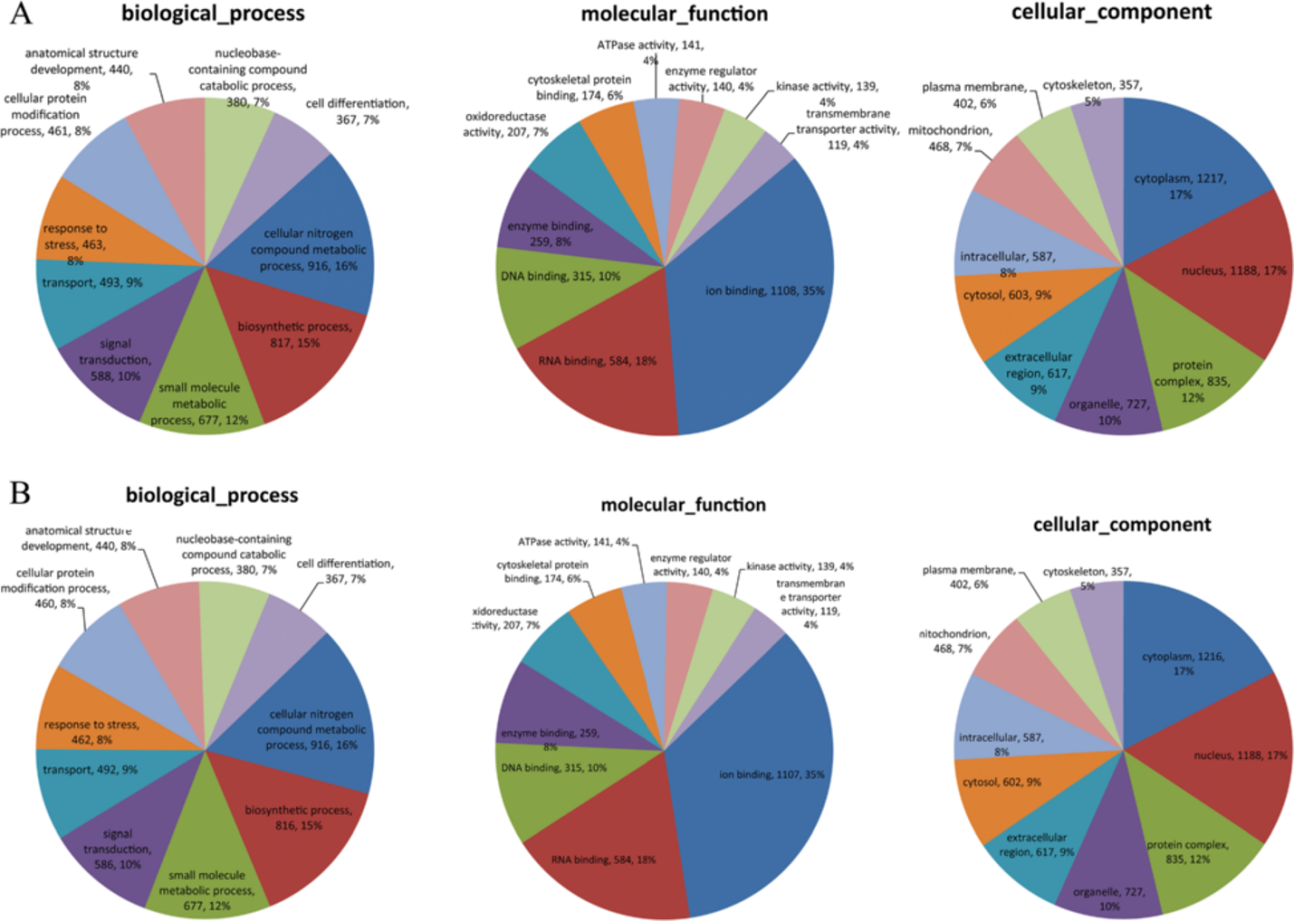
Classification of the identified proteins by GO database. **(A)**: Classification of the proteins that were identified in GMBP1-treated SGC7901/ADR cells. Biological processes (BPs), cellular components (CCs), and molecular functions (MFs) of all identified proteins, as classified according to the GO database. **(B)**: Classification of the proteins that were identified in GMBP1-treated SGC7901/VCR cells. Biological processes (BPs), cellular components (CCs), and molecular functions (MFs) of all identified proteins, as classified according to the GO database.

The functional classification of all 3,752 proteins that were identified in the GMBP1-treated SGC7901/ADR cells is presented in Figure 5A. Proteins were cataloged according to biological processes (BPs), molecular functions (MFs), and cellular components (CCs) according to the GO database. The proteins representing BPs included cellular nitrogen compound metabolic processes (16%), biosynthetic processes (15%), small molecule metabolic processes (12%), signal transduction (10%), transport (9%), response to stress (8%), cellular protein modification processes (8%), anatomical structure development (8%), nucleobase-containing compound catabolic processes (7%) and cell differentiation (7%). The MFs of the proteins were classified, and the largest groups were found to be involved in binding (77%), oxidoreductase activity (7%), ATPase activity (4%), enzyme regulator activity (4%), kinase activity (4%) and transmembrane transporter activity (4%). The proteins representing CCs were classified as cytoplasm (17%), nucleus (17%), protein complex (12%), co-organelle (10%), extracellular region (9%), cytosol (9%), intracellular (8%), mitochondrion (7%), plasma membrane (6%) and cytoskeleton (5%).

The functional classification of all 3,749 proteins identified in the GMBP1-treated SGC7901/VCR cells is presented in Figure 5B. Proteins were categorized as BPs, MFs, and CCs according to the GO database. BP proteins represented cellular nitrogen compound metabolic processes (17%), biosynthetic processes (16%), signal transduction (11%), cellular protein modification processes (9%), small molecule metabolic processes (9%), transport (8%), anatomical structure development (8%), response to stress (8%), cell differentiation (7%) and nucleobase-containing compound catabolic processes (7%). MF proteins were also classified, and the largest groups were found to be involved in binding (69%), cytoskeletal protein binding (7%), kinase activity (6%), enzyme regulator activity (6%), ATPase activity (4%), nucleic acid binding transcription factor activity (4%) and oxidoreductase activity (4%). Identified CC proteins were classified as belonging to the nucleus (19%), cytoplasm (17%), protein complex (13%), organelle (9%), intracellular (9%), extracellular region (8%), cytosol (8%), plasma membrane (6%), cytoskeleton (6%) and nucleoplasm (5%).

**Figure 6:**
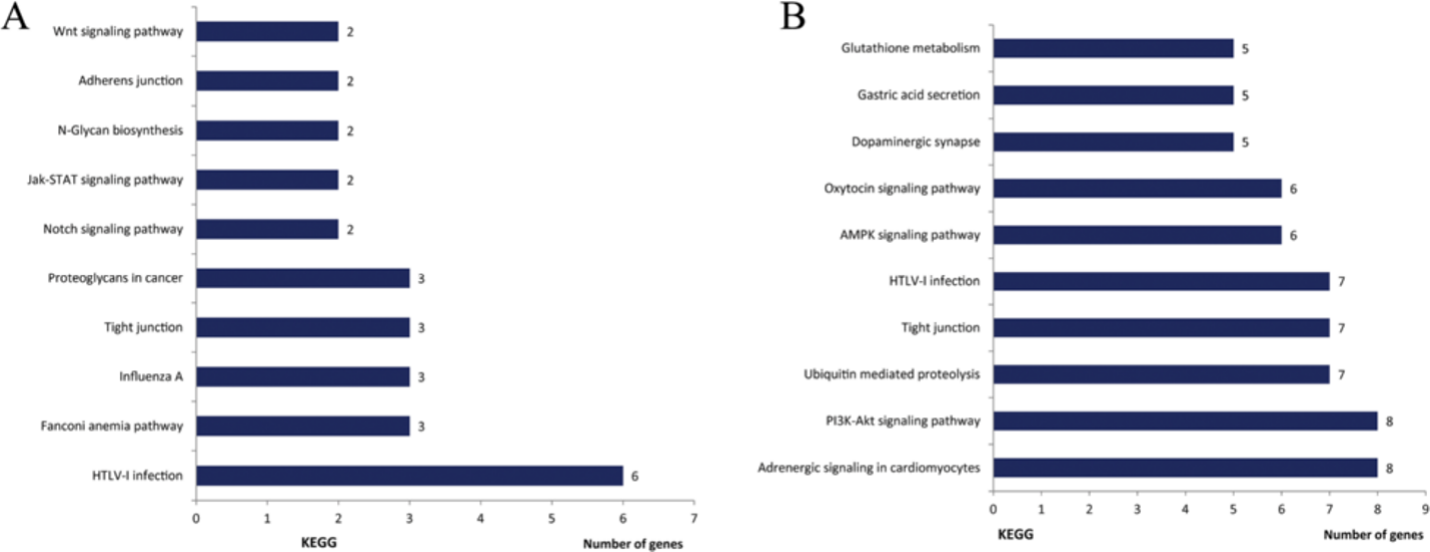
Classification of the identified proteins by KEGG database. **(A)**: The ten most significant KEGG pathways in GMBP1-treated SGC7901/ADR cells. **(B)**: The ten most significant KEGG pathways in GMBP1-treated SGC7901/VCR cells.

The differentially expressed proteins were further defined based on KEGG (http://www.genome.jp/kegg/). The proteins were mapped to KEGG pathways based on their KEGG gene ID. The proteins differentially expressed in GMBP1-treated SGC7901/ADR and SGC7901/VCR cells are involved in 38 KEGG pathways and 79 KEGG pathways, respectively (results not shown). All pathways were statistically significant and based on research. As shown in Figure 5C, we used hypergeometric distribution in the enrichment analysis to prioritize these pathways, and the top ten KEGG pathways were summarized for both cell lines. The results (Figure 6(A)) indicated ten significant (p < 0.05) pathways in the GMBP1-treated SGC7901/ADR cells, including pathways for HTLV-I infection, Fanconi anemia, Influenza A, tight junctions, proteoglycans in cancer, Notch signaling, Jak-STAT signaling, N-glycan biosynthesis, adherens junctions and Wnt signaling. Figure 6(B) shows the ten most significant pathways in the GMBP1-treated SGC7901/VCR cells, which included pathways for adrenergic signaling in cardiomyocytes, PI3K-Akt signaling, ubiquitin-mediated proteolysis, tight junctions, HTLV-I infection, AMPK signaling, oxytocin signaling, dopaminergic synapses, gastric acid secretion and glutathione metabolism. Representative pathways associated with gastric cancer were investigated, including the Notch, Wnt, p53, PI3K-Akt and calcium signaling pathways. Further research is required to verify the proposed link between these pathways and GC MDR.

### Effects of GMBP1 on several identified targets

**Figure 7:**
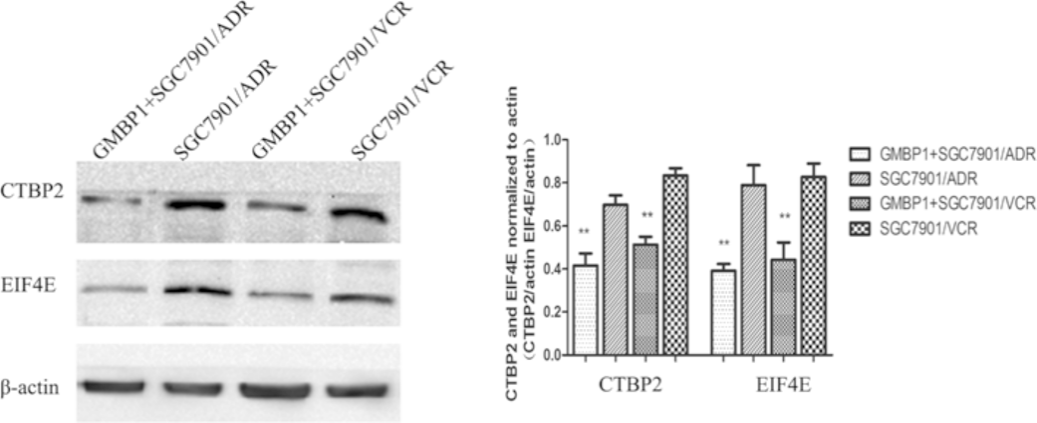
A representative western blot analysis of CTBP2 and EIF4E expression in the four cell lines comparing SGC7901/ADR and SGC7901/VCR cells and GMBP1-treated SGC7901/ADR and SGC7901/VCR cells. The values represent the means from three separate experiments, and the error bars represent the SEM (*P < 0.01).

Among the proteins that were differentially regulated in the GMBP1-treated SGC7901/ADR and SGC7901/VCR cells, those that were the most downregulated in the two cell lines, EIF4E and CTBP2, are involved in the PI3K/AKT and the Notch and Wnt signaling pathways. To validate the effects of GMBP1 on several of the identified targets, a western blotting assay was performed. As shown in Figure 7, the expression levels of EIF4E and CTBP2 proteins were clearly downregulated (p < 0.01). This trend is similar to that observed for protein expression according to the iTRAQ analysis.

## Discussion

Resistance to chemotherapy is a recurring issue for all cancer types, and the development of MDR is a major obstacle to the effective treatment of gastric cancer [33]. However, the mechanism of MDR remains obscure. To study MDR in gastric cancer, we used as cellular models two drug-resistant cell lines, SGC7901/VCR and SGC7901/ADR, which were derived from the human gastric cancer cell line SGC7901 by stepwise selection in vitro using adriamycin and vincristine, respectively. These cell lines have been widely used as in vitro models for the study of MDR in gastric cancer [34-37]. Small molecules and short peptides have been considered for use in novel research on MDR because they exhibit many advantages, including rapid blood clearance, high tissue penetration and diffusion, non-immunogenicity and a high affinity for target tumor cells [9-11]. For example, in a previous study involving many peptides, our research team identified two peptides that bind specifically to GC vascular endothelial cells: GEBP11 and GX1. GX1 was also found to inhibit tumor growth. Using a phage display approach, we investigated the GMBP1 peptide, which specifically binds to the surface of gastric cancer MDR cells and exhibits the potential to be internalized into these cells and reverse the gastric MDR phenotype. Subsequently, GRP78 was identified as a receptor for this peptide [12]. The success of our previous work and the known advantages of these short peptides encouraged us to study the effects of GMBP1 on GC MDR.

To further investigate the targeted binding sites and the subcellular localization of the GMBP1 and GRP78 peptides, we investigated the underlying internalization mechanism of GMBP1 using immunofluorescence staining combined with FACS. The results indicated the localization of GMBP1 and its receptor GRP78 in the cytoplasm of gastric cancer cells. In addition, we found that the internalization of GMBP1 into multidrug-resistant gastric cells was mediated by its receptor, GRP78. A double immunofluorescence staining assay demonstrated that the uptake of GMBP1, which was mediated by GRP78, occurred through a clathrin-independent transferrin-related pathway.

MDR is a multifactorial and multistep process, and a variety of biological factors are involved in GC MDR. Therefore, a global view of the interconnectivity of signaling proteins and their actions is critically important for the successful reversal of GC MDR. To date, proteomics analyses have proved to be powerful tools for identifying biological markers and for estimating biological networks [15]. Proteomic methods have also been used to study the mechanisms of GC MDR. For example, Hu et al. revealed that MVP, one of the differentially expressed proteins found in our study, was highly expressed in SGC7901/VCR, and MDR was verified using iTRAQ-based proteomics [26]. In the present study, the iTRAQ-based method was used to analyze the molecular mechanisms occurring in GMBP1-treated multidrug-resistant gastric cells SGC7901/ADR and SGC7901/VCR. To validate the reliability of this technology, the iTRAQ results were corroborated by conducting a literature review (in PubMed) and by western blot analysis. We determined that 83.6% of the affected proteins are also associated with other cancers, indicating that our data are consistent with those of other researchers. The differentially expressed proteins that were identified exhibited by a wide range of molecular weight (MW), pI, and GRAVY values. Moreover, bioinformatics analysis revealed that these proteins are involved in many BPs in GMBP1-treated SGC7901/ADR and SGC7901/VCR cells, including cellular nitrogen compound metabolic processes, biosynthetic processes, small molecule metabolic processes, signal transduction, transport, response to stress, cellular protein modification processes, anatomical structure development, nucleobase-containing compound catabolic processes and cell differentiation. In GMBP1-treated SGC7901/ADR cells, these proteins were found to be involved in 38 KEGG pathways that are connected with each other to form a network. Furthermore, the proteins identified in GMBP1-treated SGC7901/VCR cells are involved in 79 KEGG pathways. These findings illustrate that multiple mechanisms can cause drug resistance in gastric cancer cells and that these mechanisms might partially contribute to chemotherapeutic resistance during gastric cancer treatment.

**Figure 8:**
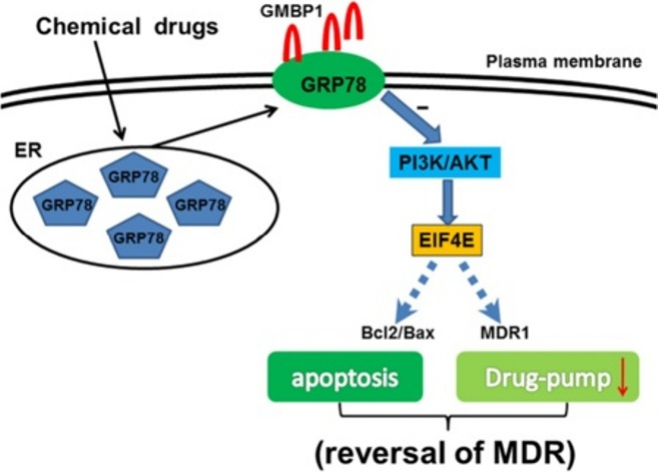
Schematic drawing of the mechanism of the GMBP1 peptide in modulating gastric cancer MDR by targeting GRP78.

Deregulation of the PI3K/AKT pathway plays a crucial role in the regulation of multiple cellular functions, including cell growth, proliferation, metabolism, and angiogenesis. Notably, numerous reports have implicated the PI3K-Akt signaling pathway in gastric cancers [38]. Among the differentially expressed proteins identified, the level of eukaryotic translation initiation factor 4E (EIF4E) was markedly downregulated in both GMBP1-treated cell lines, which was confirmed by western blot analysis. As a member of the PI3K/AKT pathway, EIF4E has been identified as an oncogene that plays a role in many malignant diseases, including GC. Silencing of EIF4E was found to slow proliferation and arrest the cell cycle in G0/G1 phase in larynx, stomach, and breast cancer cells [39-41]. These findings indicate that EIF4E expression might represent a molecular target for cancer therapy and lead to the assumption of a possible role for EIF4E in MDR. Combining our present and previous work, we hypothesis that the GMBP1 peptide modulates gastric cancer MDR by targeting GRP78 and that the expression of GRP78 modulates the expression of EIF4E and MDR1 through the PI3K/AKT pathway (as shown in Figure 8). Our study also showed that PPP2R2A, PRKAA1, and PCK2 were overexpressed in GMBP1-treated SGC7901/VCR cells. These proteins are also members of the PI3K/AKT pathway, and their function in GC MDR merits further investigation.

The Notch signaling pathway and the Wnt signaling pathway are increasingly recognized as critical for the regulation of drug resistance. C-terminal binding proteins (CTBPs) are transcriptional corepressors that mediate the Notch and Wnt pathways, among others. Of the proteins identified using iTRAQ, C-terminal binding protein 2 (CTBP2) was downregulated in both GMBP1-treated cell lines; furthermore, the protein level of CTBP2 was lower in the GMBP1-treated SGC7901/ADR and SGC7901/VCR cells than in the control cells. CTBPs interact with many DNA-binding transcription factors, including mediators of Wnt, BMP, and Notch signaling [42,43], GATA factors [44], and regulators of several key processes, including myogenesis [45], vascularization [46], apoptosis, and cell adhesion [47]. Paliwal et al. identified the CTBP2 transcription regulator as an ARF-binding protein and observed that the targeting of CTBP by ARF results in p53-independent apoptosis [48]. Furthermore, Paliwal et al. showed that CTBP might promote tumor proliferation [49]. Recent studies have shown that MDR phenotype acquisition is often associated with increased tumor invasion and metastasis [50]. MDR not only prohibits effective chemotherapy but also exacerbates the metastatic symptoms of cancer patients; CTBP proteins, which had not previously been associated with MDR, are now shown to play a role in the development of MDR.

Transcription factors and proteins related to signal transduction were found to be differentially expressed between GMBP1-treated and untreated multidrug-resistant gastric cells but have not been associated with MDR to date. The correlation between these proteins and MDR in gastric cancer cells will be the subject of future study.

## Conclusions

In conclusion, GMBP1 exhibited significant potential to reverse GC MDR. Our results showed that iTRAQ is a powerful technique for performing quantitative proteome analysis in relation to drug resistance, and a large number of differentially expressed proteins were identified in this study. Our results further confirmed that the GMBP1-GRP78 component plays an important role in the drug-resistant phenotype of gastric cancer cells. GMBP1 may therefore represent a novel MDR reversal agent for the management of GC. Other mechanisms that act against GC MDR in the GMBP1-GRP78 module should be investigated further. The data obtained will prove useful for the study of the mechanism of MDR in human GC and provide new clues for investigating MDR in other tumors.

## Additional files

Additional file 1: Table S1. iTRAQ analysis of proteins that were differentially expressed between GMBP1-treated SGC7901/ADR (iTRAQ 115) and SGC7901/ADR (iTRAQ113) cells.
Additional file 2: Table S2. iTRAQ analysis of proteins that were differentially expressed between GMBP1-treated SGC7901/VCR (iTRAQ 119) and SGC7901/VCR (iTRAQ118) cells.

## Abbreviations

GC: Gastric cancer
MDR: Multidrug resistance
FACS: Fluorescence-activated cell sorting
Itraq: Isobaric tag for relative and absolute quantitation
LC-MS: Liquid chromatography-mass spectrometry
