# Supplementary material for: Mechanism study of peptide GMBP1 and its receptor GRP78 in modulating gastric cancer MDR by iTRAQ-based proteomic analysis
Source: BMC Cancer. 2015 May 6;15:358. doi: 10.1186/s12885-015-1361-3 (PMC4430905; doi:10.1186/s12885-015-1361-3)
Supplement: Additional file 1: Table S1. — iTRAQ analysis of proteins that were differentially expressed between GMBP1-treated SGC7901/ADR (iTRAQ 115) and SGC7901/ADR (iTRAQ113) cells. [file 12885_2015_1361_MOESM1_ESM.docx]

**Table S1. iTRAQ analysis of proteins that were differentially expressed between GMBP1-treated SGC7901/ADR (iTRAQ 115) and SGC7901/ADR (iTRAQ113) cells.(143)**

| **Protein ID** | **Gene Name** | **Protein Name** | **115:113** | **MW(Da)** | **pI** | **GRAVY** |
| --- | --- | --- | --- | --- | --- | --- |
| **D6RHW1** | TAF9 | Transcription initiation factor TFIID subunit 9 | 0.557 | 11569.651 | 6.5571899 | -0.5029412 |
| **Q9UK45** | LSM7 | U6 snRNA-associated Sm-like protein LSm7 | 0.632 | 11601.764 | 5.1029663 | -0.4368932 |
| **P26447** | S100A4 CAPL MTS1 | Protein S100-A4 (Placental calcium-binding protein) | 0.59 | 11727.933 | 5.850769 | -0.5643564 |
| **P56181** | NDUFV3 | NADH dehydrogenase flavoprotein 3, mitochondrial | 0.592 | 11939.885 | 9.7201538 | -0.8601852 |
| **A0PJ62** | RPL14 | RPL14 protein | 0.604 | 14610.659 | 10.62616 | -0.6306452 |
| **Q9Y3D6** | FIS1 TTC11 CGI-135 | Mitochondrial fission 1 protein (FIS1 homolog) (hFis1) | 0.655 | 16936.78 | 8.8361206 | -0.2328947 |
| **Q6PIM8** | ACSL3 | ACSL3 protein | 0.581 | 18618.572 | 6.9264526 | -0.3743902 |
| **Q10589** | BST2 | Bone marrow stromal antigen 2 (BST-2) | 0.569 | 19767.863 | 5.4326782 | 0.0288889 |
| **H7C1U8** | APOO | Apolipoprotein O | 0.602 | 20078.855 | 8.7235718 | -0.4174157 |
| **O75223** | GGCT C7orf24 CRF21 | Gamma-glutamylcyclotransferase | 0.615 | 21006.608 | 5.0673218 | -0.4962766 |
| **H7C2Y0** | 41884 | Septin-2 | 0.658 | 21648.939 | 6.0874634 | -0.4898936 |
| **Q6PKD2** | HNRPCL1 | HNRPCL1 protein | 0.63 | 22650.658 | 9.9454956 | -0.631401 |
| **A2AAT0** | SLC39A7 | Zinc transporter SLC39A7 | 0.581 | 24001.865 | 9.8015747 | -0.5604545 |
| **P82930** | MRPS34 | 28S ribosomal protein S34, mitochondrial | 0.568 | 25649.142 | 9.982605 | -0.7674312 |
| **Q69YV6** | DKFZp564G1016 | Putative uncharacterized protein DKFZp564C0716 | 0.665 | 27637.43 | 8.9321899 | -0.1485714 |
| **Q8N129** | CNPY4 | Protein canopy homolog 4 | 0.156 | 28308.24 | 4.5964966 | -0.6967742 |
| **A6NCD4** | C10orf131 | Uncharacterized protein C10orf131 | 0.606 | 29613.328 | 5.9711304 | -0.7679687 |
| **K7EQ91** | SLC39A6 | Zinc transporter ZIP6 | 0.365 | 30911.191 | 5.8449097 | -0.3981685 |
| **F2Z393** | TALDO1 | Transaldolase | 0.647 | 35326.933 | 9.0747681 | -0.1814465 |
| **Q9Y2S2** | CRYL1 CRY | Lambda-crystallin homolog | 0.61 | 35417.343 | 5.8070679 | -0.0407524 |
| **Q9Y673** | ALG5 HSPC149 | Dolichyl-phosphate beta-glucosyltransferase | 0.614 | 36944.167 | 9.3355103 | -0.0907407 |
| **Q01664** | TFAP4 BHLHC41 | Transcription factor AP-4 | 0.614 | 38723.575 | 5.6288452 | -0.8970414 |
| **Q8N8C0** | ZNF781 | Zinc finger protein 781 | 0.509 | 41524.137 | 10.395081 | -0.0616901 |
| **Q9BYX7** | POTEKP ACTBL3 FKSG30 | Putative beta-actin-like protein 3 | 0.314 | 42014.026 | 5.9130249 | -0.2789333 |
| **A6NGH8** | ANKRD61 | Ankyrin repeat domain-containing protein 61 | 0.621 | 46138.05 | 9.1036987 | -0.0648325 |
| **P56545** | CTBP2 | C-terminal-binding protein 2 (CtBP2) | 0.614 | 48942.207 | 6.4712524 | -0.2229213 |
| **Q9BQ95** | ECSIT | Evolutionarily conserved signaling intermediate in Toll pathway, | 0.603 | 49145.524 | 5.8927612 | -0.4464037 |
| **Q96SQ9** | CYP2S1 | Cytochrome P450 2S1 | 0.558 | 55813.623 | 8.796814 | -0.0244048 |
| **Q9BYC5** | FUT8 | Alpha-(1,6)-fucosyltransferase | 0.649 | 66512.261 | 7.3632202 | -0.5511304 |
| **Q86X52** | CHSY1 | Chondroitin sulfate synthase 1 | 0.607 | 91779.572 | 9.3080444 | -0.4428928 |
| **O95263** | PDE8B PIG22 | High affinity cAMP-specific and IBMX-insensitive 3',5'-cyclic phosphodiesterase 8B (HsPDE8B) | 0.356 | 98973.655 | 6.3527222 | -0.3787571 |
| **Q6NUP7** | PPP4R4 KIAA1622 PP4R4 | Serine/threonine-protein phosphatase 4 regulatory subunit 4 | 0.604 | 99446.74 | 7.9556274 | -0.2756014 |
| **Q86VM9** | ZC3H18 NHN1 | Zinc finger CCCH domain-containing protein 18 | 0.67 | 106372.72 | 8.3949585 | -1.5887723 |
| **P52333** | JAK3 | Tyrosine-protein kinase JAK3 (EC 2.7.10.2) (Janus kinase 3) | 0.492 | 125092.18 | 6.769104 | -0.1475979 |
| **O75094** | SLIT3 | Slit homolog 3 protein (Slit-3) | 0.63 | 167704.56 | 7.9921265 | -0.2465529 |
| **P51531** | SMARCA2 | ATP-dependent helicase SMARCA2 | 0.56 | 181269.82 | 6.7567749 | -0.8950943 |
| **Q8IZD9** | DOCK3 KIAA0299 MOCA | Dedicator of cytokinesis protein 3 (Modifier of cell adhesion) | 0.657 | 233090.95 | 6.5181274 | -0.3589655 |
| **P06730** | EIF4E | Eukaryotic translation initiation factor 4E | 0.668 | 25097.25 | 6.031 | -0.696774 |
| **C0JYY2** | APOB hCG_20898 | Apolipoprotein B (Including Ag(X) antigen) | 0.598 | 515529.74 | 6.5839233 | -0.2962525 |
| **Q8WWX9** | SELM SEPM | Selenoprotein M (SelM) | 0.641 | 16231.66 | 5.2401 | -0.127 |
| **A1C2E7** | GDF8 | Myostatin | 0.399 | 42776.38 | 6.4817 | -0.39 |
| **U3KQC1** | WDR18 | WD repeat-containing protein 18 | 0.561 | 43356.25 | 6.8393 | -0.087 |
| **E9PH24** | GRID2 GLURD2 | Glutamate receptor ionotropic, delta-2 (GluD2) | 0.661 | 113356.1 | 5.73 | -0.193 |
| **H0Y412** | PRRC2B | Protein PRRC2B | 0.537 | 63316.42 | 6.6777 | -0.966 |
| **K7EMN4** | APLP1 | Amyloid-like protein 1 | 0.612 | 24496.29 | 5.8104 | -0.695 |
| **M0R0B4** | KXD1 | KxDL motif-containing protein 1 | 0.633 | 9950.29 | 11.345 | -0.475 |
| **H0Y539** | SHC1 | SHC-transforming protein 1 | 0.557 | 32625.87 | 6.9148 | -0.415 |
| **H0YB01** | EXTL3 | Exostosin-like 3 | 0.595 | 8505.52 | 6.2011 | -0.065 |
| **D6R9Z7** | COX7C | Cytochrome c oxidase subunit 7C, mitochondrial | 1.794 | 6382.1372 | 9.857971 | 0.064285714 |
| **Q96B49** | TOM6 | Mitochondrial import receptor subunit TOM6 homolog | 1.663 | 8001.5927 | 4.663757 | -0.021621622 |
| **F8WEP0** | EXOG | Nuclease EXOG, mitochondrial | 1.759 | 8197.7848 | 9.684753 | 0.148148148 |
| **H0Y4T6** | PIN4 | Peptidyl-prolyl cis-trans isomerase NIMA-interacting 4 | 1.696 | 9542.4009 | 10.17499 | -0.913186813 |
| **Q7Z4G1** | COMMD6 | COMM domain-containing protein 6 | 1.522 | 9637.6021 | 5.69281 | -0.165882353 |
| **Q15651** | HMGN3 | High mobility group nucleosome-binding domain-containing protein 3 | 1.622 | 10665.2636 | 9.66156 | -1.828282828 |
| **Q9GZP8** | IMUP C19orf33 | Immortalization up-regulated protein(H2RSP) | 9.293 | 10896.444 | 9.730896 | -1.371698113 |
| **P07311** | ACYP1 ACYPE | Acylphosphatase-1 | 1.516 | 11260.2441 | 9.300598 | -0.556565657 |
| **J3QRX6** | COPRS | Coordinator of PRMT5 and differentiation stimulator | 1.609 | 11418.5285 | 3.809143 | -1.130097087 |
| **Q96DE5** | ANAPC16 | Anaphase-promoting complex subunit 16 (APC16) | 1.582 | 11666.3924 | 4.912659 | -0.228181818 |
| **E9PMU3** | EED | Polycomb protein EED | 1.676 | 12448.5872 | 5.849426 | -0.399056604 |
| **E9PRR7** | FRG1 | Protein FRG1 | 1.9 | 12515.4228 | 5.136047 | -0.431034483 |
| **O60739** | EIF1B | Eukaryotic translation initiation factor 1b (eIF1b) | 1.513 | 12822.9588 | 6.822327 | -0.527433628 |
| **I3L464** | NDE1 | Nuclear distribution protein nudE homolog 1 | 1.516 | 12989.6553 | 4.835999 | -1.176146789 |
| **M0QZ21** | AP2S1 | AP-2 complex subunit sigma | 1.752 | 14555.9456 | 4.927429 | -0.042622951 |
| **K7EKA0** | FOSB | Protein fosB | 1.895 | 14738.6253 | 10.23322 | -0.908029197 |
| **K7ENJ0** | RAD23A | UV excision repair protein RAD23 homolog A | 2.124 | 14853.9831 | 5.485168 | -0.538129496 |
| **O60361** | NME2P1 | Putative nucleoside diphosphate kinase (NDK) | 2.594 | 15528.2493 | 8.759949 | -0.277372263 |
| **Q9NRF9** | POLE3 CHRAC17 | DNA polymerase epsilon subunit 3 | 1.507 | 16858.6927 | 4.678894 | -1.202040816 |
| **O75575** | CRCP | DNA-directed RNA polymerase III subunit RPC9 | 1.786 | 16870.1175 | 5.292664 | -0.745945946 |
| **H0YE54** | GOLGA1 | Golgin subfamily A member 1 | 1.502 | 17396.4802 | 5.117981 | -0.869536424 |
| **Q1A5X7** | WHAMMP3 WHAMML1 | Putative WASP homolog-associated protein with actin, membranes and microtubules-like protein 1 | 1.541 | 18090.3038 | 5.711121 | -0.390849673 |
| **P49006** | MARCKSL1 MLP | MARCKS-related protein (MARCKS-like protein 1) | 1.672 | 19527.6491 | 4.680237 | -1.158461538 |
| **O00762** | UBE2C UBCH10 | Ubiquitin-conjugating enzyme E2 C (UbcH10) | 1.587 | 19651.2319 | 6.827454 | -0.393296089 |
| **E9PQX9** | DRAP1 | Dr1-associated corepressor | 1.608 | 19964.7797 | 4.50885 | -0.952972973 |
| **Q0VDK5** | OTUD3 | OTUD3 protein | 1.902 | 20003.9701 | 6.015808 | -1.152542373 |
| **B7Z8B3** | PEX19 | Peroxisomal biogenesis factor 19 | 1.787 | 20119.9591 | 5.042542 | -0.776344086 |
| **Q5JPE4** | DKFZp667O202 | Putative uncharacterized protein DKFZp667O202 | 1.641 | 20391.4703 | 6.286072 | -0.033149171 |
| **P18847** | ATF3 | Cyclic AMP-dependent transcription factor ATF-3 | 1.694 | 20574.5547 | 8.802551 | -0.671270718 |
| **Q8IXQ3** | C9orf40 | Uncharacterized protein C9orf40 | 1.65 | 21062.1524 | 4.886414 | -0.884536082 |
| **P05090** | APOD | Apolipoprotein D (Apo-D) (ApoD) | 2.393 | 21274.4249 | 5.058289 | -0.053439153 |
| **B7Z4J8** | ZNF346 | Zinc finger protein 346 | 1.621 | 21709.4571 | 8.856506 | -0.647959184 |
| **C9JTS3** | AAMP | Angio-associated migratory cell protein | 1.658 | 21991.1275 | 4.169128 | -0.397 |
| **Q14116** | IL18 IGIF IL1F4 | Interleukin-18 (IL-18) | 1.5 | 22325.0986 | 4.538879 | -0.479274611 |
| **Q9NPD8** | UBE2T HSPC150 | Ubiquitin-conjugating enzyme E2 T ( | 1.549 | 22519.6737 | 7.775208 | -0.682741117 |
| **D6RFL4** | CD14 | Monocyte differentiation antigen CD14, urinary form | 2.349 | 23311.6717 | 5.140198 | 0.066197183 |
| **P62070** | RRAS2 TC21 | Ras-related protein R-Ras2 (Ras-like protein TC21) | 1.777 | 23398.352 | 5.736389 | -0.549019608 |
| **C9J8H1** | ATP6V1E1 | V-type proton ATPase subunit E 1 | 1.534 | 23538.1481 | 9.111389 | -0.513793103 |
| **F5GYH0** | GSG1 | Germ cell-specific gene 1 protein | 1.737 | 23593.1196 | 8.604675 | -0.225592417 |
| **Q6P5S8** | IGK@ | IGK@ protein | 3.969 | 25771.448 | 5.942932 | -0.288559322 |
| **C9J306** | HACL1 | 2-hydroxyacyl-CoA lyase 1 | 1.667 | 26387.0615 | 8.109192 | 0.048360656 |
| **Q96CF2** | CHMP4C SHAX3 | Charged multivesicular body protein 4c | 1.607 | 26409.2863 | 5.832825 | -0.924034335 |
| **B4DUW3** | LCMT2 | cDNA FLJ51321, highly similar to Leucine carboxyl methyltransferase 2 | 1.994 | 29022.4381 | 5.794006 | 0.043396226 |
| **P25786** | PSMA1 HC2 NU | Proteasome subunit alpha type-1 | 1.521 | 29554.035 | 6.147644 | -0.433079848 |
| **Q9UBV8** | PEF1 ABP32 | Peflin | 1.985 | 30379.2241 | 6.097229 | -0.53028169 |
| **Q5JUA9** | VPS16 | Vacuolar protein sorting-associated protein 16 homolog | 2.218 | 30406.7917 | 8.723572 | 0.092335766 |
| **B4DMI0** | FAM172A | Protein FAM172A (cDNA FLJ51952) | 1.662 | 31370.1625 | 6.771179 | -0.60929368 |
| **Q9NPE2** | NGRN FI58G HT020 | Neugrin (Mesenchymal stem cell protein DSC92) | 1.984 | 32405.8419 | 9.162415 | -0.73814433 |
| **Q9NVM6** | DNAJC17 | DnaJ homolog subfamily C member 17 | 2.865 | 34685.493 | 8.60907 | -0.971052632 |
| **B4DZP2** | TCF12 | Transcription factor 12 | 1.635 | 34690.3316 | 6.27948 | -0.933227848 |
| **Q6N039** | TMCC1 | Transmembrane and coiled-coil domains protein 1 | 1.714 | 36519.1773 | 5.164001 | -0.269300912 |
| **Q29939** | HLA-B | MHC class I lymphocyte antigen | 1.566 | 39211.4236 | 5.927429 | -0.636494253 |
| **Q6P1K9** | PANK2 | PANK2 protein | 2.083 | 42327.2623 | 5.558167 | -0.014507772 |
| **H0YIV4** | NAP1L1 | Nucleosome assembly protein 1-like 1 | 2.007 | 44712.0951 | 4.383972 | -0.961558442 |
| **P14324** | FDPS FPS KIAA1293 | Farnesyl pyrophosphate synthase (FPP synthase) | 1.728 | 48272.9366 | 5.833069 | -0.237470167 |
| **P55010** | EIF5 | Eukaryotic translation initiation factor 5 (eIF-5) | 1.58 | 49220.1435 | 5.411682 | -0.802088167 |
| **Q6FI91** | TSPYL | TSPYL protein | 1.577 | 49303.9515 | 5.470032 | -0.645890411 |
| **O15427** | SLC16A3 MCT4 | Monocarboxylate transporter 4 (MCT 4) | 2.05 | 49466.6234 | 8.22699 | 0.66172043 |
| **Q6IQ49** | SDE2 C1orf55 | Protein SDE2 homolog | 1.524 | 49739.3399 | 5.773254 | -0.717960089 |
| **Q9BQE3** | TUBA1C TUBA6 | Tubulin alpha-1C chain (Alpha-tubulin 6) | 1.524 | 49892.6623 | 4.964172 | -0.23363029 |
| **D6RBU5** | NFASC | Neurofascin | 2.628 | 54474.2389 | 8.680725 | -0.441152263 |
| **Q9UNF0** | PACSIN2 | Protein kinase C and casein kinase substrate in neurons protein 2 | 1.525 | 55735.7154 | 5.083679 | -1.02962963 |
| **B5MBX1** | JADE2 | Protein Jade-2 | 2.405 | 57374.995 | 5.318176 | -0.609626719 |
| **Q5T376** | FRMD4A | FERM domain containing 4A, isoform CRA_c | 2.058 | 60198.1595 | 8.981018 | -0.386666667 |
| **Q7Z3K6** | MIER3 | Mesoderm induction early response protein 3 (Mi-er3) | 2.882 | 61433.2817 | 4.417664 | -0.618909091 |
| **P40222** | TXLNA TXLN | Alpha-taxilin | 1.504 | 61887.8495 | 6.148254 | -1.232417582 |
| **J3KP30** | DNTTIP2 | Deoxynucleotidyltransferase terminal-interacting protein 2 | 1.734 | 66171.3661 | 5.003357 | -0.964725458 |
| **Q9H9A7** | RMI1 C9orf76 | RecQ-mediated genome instability protein 1 | 1.787 | 70140.6986 | 4.85907 | -0.38352 |
| **P02545** | LMNA LMN1 | Prelamin-A/C [Cleaved into: Lamin-A/C (70 kDa lamin) | 0.968 | 74135.4998 | 6.569031 | -0.862951807 |
| **Q92581** | SLC9A6 KIAA0267 | Sodium/hydrogen exchanger 6 (Na(+)/H(+) exchanger 6) | 1.904 | 74157.6128 | 6.026672 | 0.277428999 |
| **Q05D30** | CLSPN | CLSPN protein | 1.561 | 74288.3155 | 5.278503 | -1.18100304 |
| **A6NN80** | ANXA6 hCG_39152 | Annexin | 1.75 | 75272.5497 | 5.460144 | -0.474512744 |
| **Q96T51** | RUFY1 RABIP4 | RUN and FYVE domain-containing protein 1 | 1.731 | 79813.7017 | 5.542542 | -0.626694915 |
| **Q05BX6** | RABEP1 | RABEP1 protein | 2.335 | 82373.0309 | 5.056702 | -0.893715084 |
| **B7WP74** | CWC22 | Pre-mRNA-splicing factor CWC22 homolog | 1.566 | 85572.3433 | 5.513733 | -0.808187919 |
| **E7EWK3** | DHX36 | ATP-dependent RNA helicase DHX36 | 1.933 | 91426.2736 | 6.889221 | -0.387578419 |
| **E9PR38** | PUM1 | Pumilio homolog 1 | 2.117 | 100494.306 | 8.696106 | -0.293432203 |
| **Q8TCG1** | KIAA1524 CIP2A | Protein CIP2A (Cancerous inhibitor of PP2A) | 1.626 | 102179.576 | 5.84967 | -0.187513812 |
| **P26232** | CTNNA2 CAPR | Catenin alpha-2 (Alpha N-catenin) | 1.754 | 105307.302 | 5.520447 | -0.341972718 |
| **Q15042** | RAB3GAP1 | Rab3 GTPase-activating protein catalytic subunit | 1.632 | 110517.687 | 5.375549 | -0.440672783 |
| **Q9P2R3** | ANKFY1 | Ankyrin repeat and FYVE domain-containing protein 1 | 1.625 | 128392.287 | 5.703674 | -0.154491018 |
| **B7ZLE1** | FRMPD4 | FRMPD4 protein | 1.66 | 143519.541 | 5.138611 | -0.482724505 |
| **Q7Z6C1** | EP300 | EP300 protein | 2.168 | 170072.401 | 7.356384 | -0.694462331 |
| **Q9BXT8** | RNF17 TDRD4 | RING finger protein 17 (Tudor domain-containing protein 4) | 2.262 | 184633.77 | 5.272644 | -0.372396796 |
| **Q8NI35** | INADL PATJ | InaD-like protein (Inadl protein) (hINADL) | 1.626 | 196357.253 | 4.836975 | -0.40294281 |
| **Q12873** | CHD3 | Chromodomain-helicase-DNA-binding protein 3 (CHD-3) | 1.558 | 226579.641 | 6.921326 | -0.812 |
| **Q86YA3** | ZGRF1 C4orf21 | Protein ZGRF1 | 2.054 | 236589.018 | 5.807556 | -0.497290875 |
| **B4DEM2** | DBNL | Drebrin-like protein | 1.526 | 48207.28 | 5.01 | -0.844 |
| **H0YCX3** | SLC24A4 | Sodium/potassium/calcium exchanger 4 | 1.553 | 51220.5 | 51220.5 | 0.312 |
| **J3KSZ6** | TERF2 | Telomeric repeat-binding factor 2 | 1.682 | 15474.45 | 15474.45 | -0.82 |
| **H0YA55** | ALB | Serum albumin | 1.523 | 51578.23 | 51578.23 | -0.353 |
| **H0Y750** | ATP6AP2 | Renin receptor | 1.543 | 26640.56 | 26640.56 | 0.043 |
| **H0Y8F2** | TACC3 | Transforming acidic coiled-coil-containing protein 3 | 1.592 | 23232.4 | 23232.4 | -0.838 |
| **H7BZH3** | PEX1 | Peroxisome biogenesis factor 1 | 1.53 | 22653.92 | 22653.92 | -0.418 |
| **H3BQJ1** | ATP6V0D1 | V-type proton ATPase subunit d 1 | 1.537 | 25646.17 | 25646.17 | -0.027 |
