# Supplementary material for: Mechanism study of peptide GMBP1 and its receptor GRP78 in modulating gastric cancer MDR by iTRAQ-based proteomic analysis
Source: BMC Cancer. 2015 May 6;15:358. doi: 10.1186/s12885-015-1361-3 (PMC4430905; doi:10.1186/s12885-015-1361-3)
Supplement: Additional file 2: Table S2. — iTRAQ analysis of proteins that were differentially expressed between GMBP1-treated SGC7901/VCR (iTRAQ 119) and SGC7901/VCR (iTRAQ118) cells. [file 12885_2015_1361_MOESM2_ESM.docx]

**Table S2. iTRAQ analysis of proteins that were differentially expressed between GMBP1-treated SGC7901/VCR (iTRAQ 119) and SGC7901/VCR (iTRAQ118) cells.(217)**

| **Protein ID** | **Gene Name** | **Protein Name** | **119:118** | **MW(Da)** | **pI** | **GRAVY** |
| --- | --- | --- | --- | --- | --- | --- |
| Q15651 | HMGN3 | High mobility group nucleosome-binding domain-containing protein 3 | 0.431 | 10665.85 | 10.4585 | -1.828283 |
| P56545 | CTBP2 | C-terminal-binding protein 2 (CtBP2) | 0.657 | 48942.21 | 6.4712524 | -1.222921 |
| C9J813 | CALD1 | Caldesmon | 0.66 | 54158.76 | 5.3952 | -1.682135 |
| Q9Y2W1 | THRAP3 | Thyroid hormone receptor-associated protein 3 | 0.563 | 108665.78 | 10.82 | -1.628168 |
| Q5STZ8 | ABCF1 | ATP-binding cassette sub-family F member 1 | 0.478 | 38318.72 | 5.5724 | -1.547493 |
| Q96MU7 | YTHDC1 | YTH domain-containing protein 1 | 0.67 | 84700.34 | 6.1308 | -1.518569 |
| Q5JSL0 | HMGN5 | High mobility group nucleosome-binding domain-containing protein 5 | 0.617 | 16496.08 | 4.3824 | -1.421622 |
| Q9H1J1 | UPF3A | Regulator of nonsense transcripts 3A | 0.664 | 54696.24 | 9.5091 | -1.364286 |
| K7ELS0 | C19orf43 | Uncharacterized protein C19orf43 | 0.651 | 13503.28 | 4.5641 | -1.347727 |
| Q01105 | SET | Protein SET | 0.603 | 33488.88 | 3.9497 | -1.333793 |
| K7ELA4 | CBX1 | Chromobox protein homolog 1 | 0.459 | 15980.87 | 4.6721 | -1.281159 |
| Q05DQ2 | RNMT | RNMT protein | 0.648 | 25406.71 | 9.8671 | -1.204444 |
| Q05D30 | CLSPN | CLSPN protein | 0.573 | 74292.29 | 5.0478 | -1.181003 |
| I3L464 | NDE1 | Nuclear distribution protein nudE homolog 1 | 0.646 | 12990.31 | 4.512 | -1.176147 |
| P49006 | MARCKSL1 | MARCKS-like protein 1 | 0.514 | 19528.8 | 4.3384 | -1.158462 |
| Q0VDK5 | OTUD3 | OTUD3 protein | 0.334 | 20005.02 | 6.2673 | -1.152542 |
| A7BI36 | RRBP1 | p180/ribosome receptor | 0.504 | 165748.47 | 9.6721 | -1.148117 |
| Q01844 | EWSR1 EWS | RNA-binding protein EWS | 0.489 | 68478.24 | 9.5589 | -1.116921 |
| Q96EU6 | RRP36 | Ribosomal RNA processing protein 36 homolog | 0.508 | 29823.11 | 10.868 | -1.113127 |
| Q4LE39 | ARID4B | AT-rich interactive domain-containing protein 4B | 0.344 | 147809.49 | 4.7623 | -1.093293 |
| Q9UHB6 | LIMA1 | LIM domain and actin-binding protein 1 | 0.374 | 85225.55 | 6.8208 | -1.073781 |
| Q14241 | TCEB3 | Transcription elongation factor B polypeptide 3 | 0.473 | 89908.83 | 10.2139 | -1.067419 |
| H3BV63 | CCPG1 | Cell cycle progression protein 1 | 0.551 | 13775.25 | 9.3339 | -1.031 |
| H7C3H4 | SETD2 | Histone-lysine N-methyltransferase SETD2 | 0.507 | 189680.06 | 5.3101 | -1.028 |
| P48634 | PRRC2A | Protein PRRC2A | 0.429 | 228863.19 | 9.9985 | -1.027816 |
| Q86V48 | LUZP1 | Leucine zipper protein 1 | 0.573 | 120274.88 | 8.7937 | -1.025465 |
| Q15545 | TAF7 | Transcription initiation factor TFIID subunit 7 | 0.649 | 40259.07 | 4.833 | -1.008023 |
| Q8NFC6 | BOD1L1 | Biorientation of chromosomes in cell division protein 1-like 1 | 0.669 | 330466.44 | 4.729 | -0.9902 |
| Q00839 | HNRNPU | Heterogeneous nuclear ribonucleoprotein U | 0.591 | 90584.43 | 5.8016 | -0.977576 |
| Q9NVM6 | DNAJC17 | DnaJ homolog subfamily C member 17 | 0.358 | 34687.33 | 8.852 | -0.971053 |
| A0MZ66 | KIAA1598 | Shootin-1 | 0.638 | 71639.86 | 5.0058 | -0.968621 |
| H0YIV4 | NAP1L1 | Nucleosome assembly protein 1-like 1 | 0.545 | 44714.44 | 4.1118 | -0.961558 |
| H0YNN5 | LACTB | Serine beta-lactamase-like protein LACTB | 0.469 | 14617.89 | 9.8885 | -0.955556 |
| E9PQX9 | DRAP1 | Dr1-associated corepressor | 0.646 | 19965.87 | 4.2592 | -0.952973 |
| H0YG85 | RASSF8 | Ras association domain-containing protein 8 | 0.577 | 23944.88 | 4.6149 | -0.946154 |
| Q9BVJ6 | UTP14A | U3 small nucleolar RNA-associated protein 14 homolog A | 0.347 | 87977.86 | 7.9394 | -0.941245 |
| Q92576 | PHF3 | PHD finger protein 3 | 0.559 | 229481.29 | 6.9442 | -0.939333 |
| B4DZP2 | TCF12 | Transcription factor 12 | 0.473 | 34692.2 | 6.7418 | -0.933228 |
| F8VRS2 | CNOT2 | CCR4-NOT transcription complex subunit 2 | 0.581 | 16669.06 | 5.1267 | -0.924204 |
| H0Y4T6 | PIN4 | Peptidyl-prolyl cis-trans isomerase NIMA-interacting 4 | 0.42 | 9542.92 | 10.9255 | -0.913187 |
| Q9H788 | SH2D4A | SH2 domain-containing protein 4A | 0.655 | 52726.99 | 8.1269 | -0.90859 |
| Q9NYV4 | CDK12 | Cyclin-dependent kinase 12 | 0.554 | 164154.6 | 10.0029 | -0.908121 |
| K7EKA0 | FOSB | Protein fosB | 0.626 | 14739.43 | 10.7092 | -0.908029 |
| C9J4K0 | C2orf49 | Ashwin | 0.608 | 21356.48 | 10.4255 | -0.899474 |
| Q9NXT0 | ZNF586 | Zinc finger protein 586 | 0.414 | 46413.23 | 8.9496 | -0.897761 |
| A5PLN4 | SF4 | Splicing factor 4 | 0.6 | 72529.93 | 7.9462 | -0.895039 |
| E9PKQ0 | STRBP | Spermatid perinuclear RNA-binding protein | 0.465 | 8400.26 | 4.6485 | -0.894595 |
| O43583 | DENR | Density-regulated protein | 0.645 | 22092.01 | 4.9278 | -0.707576 |
| E7EWR7 | NELFE | Negative elongation factor E | 0.513 | 6579.64 | 8.0185 | -0.703509 |
| P06730 | EIF4E | Eukaryotic translation initiation factor 4E | 0.668 | 25097.25 | 6.031 | -0.696774 |
| P11532 | DMD | Dystrophin | 0.61 | 426749.62 | 5.6885 | -0.694817 |
| Q7Z6C1 | EP300 | EP300 protein | 0.334 | 170081.44 | 7.5039 | -0.694462 |
| Q3KR37 | GRAMD1B | GRAM domain-containing protein 1B | 0.38 | 85400.09 | 6.1338 | -0.689973 |
| M0R3D6 | RPL18A | 60S ribosomal protein L18a | 0.622 | 16714.54 | 11.3823 | -0.685106 |
| E9PFK5 | NOP14 | Nucleolar protein 14 | 0.643 | 88866.01 | 6.1059 | -0.676904 |
| F8WBS5 | RPL35A | 60S ribosomal protein L35a | 0.479 | 6472.54 | 10.5062 | -0.676364 |
| F8W0J4 | YEATS4 | YEATS domain-containing protein 4 | 0.638 | 20131.13 | 9.1723 | -0.671098 |
| P52907 | CAPZA1 | F-actin-capping protein subunit alpha-1 | 0.439 | 32922.77 | 5.4219 | -0.668182 |
| D6REL5 | FAM175A | BRCA1-A complex subunit Abraxas | 0.589 | 41578.19 | 8.1796 | -0.6575 |
| Q6FI91 | TSPYL | TSPYL protein | 0.49 | 49306.59 | 5.3307 | -0.64589 |
| O76094 | SRP72 | Signal recognition particle subunit SRP72 | 0.609 | 74606.2 | 9.7978 | -0.642027 |
| Q13409 | DYNC1I2 | Cytoplasmic dynein 1 intermediate chain 2 | 0.558 | 71456.59 | 4.853 | -0.637931 |
| Q29939 | HLA-B | MHC class I lymphocyte antigen | 0.466 | 39213.54 | 6.3398 | -0.636494 |
| E9PIH6 | EHBP1L1 | EH domain-binding protein 1-like protein 1) | 0.427 | 71673.01 | 5.5266 | -0.634925 |
| Q9H1X3 | DNAJC25 | DnaJ homolog subfamily C member 25 | 0.617 | 42403.81 | 9.4783 | -0.632778 |
| Q9NYL2 | HCCS4 | Human cervical cancer suppressor gene 4 protein | 0.525 | 91155.02 | 7.8754 | -0.628375 |
| Q96IL1 | DIAPH1 | DIAPH1 protein | 0.493 | 52118.71 | 6.5409 | -0.624123 |
| G3V5X4 | SYNE2 | Nesprin-2 | 0.448 | 787723.06 | 5.1397 | -0.622382 |
| E9PL01 | SPCS2 | Signal peptidase complex subunit 2 | 0.587 | 17027.19 | 9.4753 | -0.620382 |
| Q86UV5 | USP48 | Ubiquitin carboxyl-terminal hydrolase 48 | 0.539 | 119032.28 | 5.9144 | -0.62029 |
| Q7Z3K6 | MIER3 | Mesoderm induction early response protein 3 | 0.134 | 61436.53 | 4.1402 | -0.618909 |
| Q6NTA2 | HNRNPL | HNRNPL protein | 0.64 | 61927.12 | 7.3577 | -0.615909 |
| Q9BVM2 | DPCD | Protein DPCD | 0.547 | 23239.75 | 9.4667 | -0.614778 |
| F8W822 | GIT2 | ARF GTPase-activating protein GIT2 | 0.624 | 46988.6 | 6.7771 | -0.613302 |
| Q58F08 | ANKIB1 | ANKIB1 protein | 0.62 | 80955.32 | 4.6696 | -0.613122 |
| B5MBX1 | JADE2 | Protein Jade-2 | 0.632 | 57377.98 | 5.1062 | -0.609627 |
| E9PB39 | ARHGEF10 | Rho guanine nucleotide exchange factor 10 | 0.513 | 68222.44 | 4.3619 | -0.608361 |
| P31943 | HNRPH | Heterogeneous nuclear ribonucleoprotein H | 0.526 | 49229.47 | 6.2393 | -0.602227 |
| A9QQ22 | WASH | Actin nucleation promoting factor | 0.582 | 43822.51 | 5.2397 | -0.585122 |
| K7ESL1 | TXNL4A | Thioredoxin-like protein 4A | 0.634 | 8462.76 | 8.8283 | -0.577465 |
| A4FUT8 | JMJD1B | JMJD1B protein | 0.665 | 169055.04 | 7.5201 | -0.574984 |
| Q5BKY2 | EIF3H | Eukaryotic translation initiation factor 3 subunit H | 0.62 | 39609.84 | 6.3463 | -0.574785 |
| F8W8I6 | TIA1 | Nucleolysin TIA-1 isoform p40 | 0.63 | 42835.01 | 7.6896 | -0.570649 |
| Q8N392 | ARHGAP18 | Rho GTPase-activating protein 18 | 0.403 | 74976.74 | 6.3586 | -0.560181 |
| P07311 | ACYP1 | Acylphosphatase-1 | 0.644 | 11260.85 | 9.9296 | -0.556566 |
| Q9H8G2 | CAAP1 | Caspase activity and apoptosis inhibitor 1 | 0.303 | 38367.86 | 4.3445 | -0.548199 |
| P49207 | RPL34 | 60S ribosomal protein L34 | 0.584 | 13292.95 | 12.0188 | -0.541026 |
| Q12789 | GTF3C1 | General transcription factor 3C polypeptide 1 | 0.533 | 238874.78 | 7.2901 | -0.538644 |
| H3BRP2 | TGFB1I1 | Transforming growth factor beta-1-induced transcript 1 protein | 1.904 | 5584.201 | 8.8851929 | -0.044231 |
| H3BUD2 | DCTN5 | Dynactin subunit 5 | 2.145 | 6023.667 | 7.6289673 | -0.037736 |
| I3L407 | FN3KRP | Ketosamine-3-kinase | 1.527 | 6036.45 | 8.8014526 | -0.632143 |
| B7ZBQ1 | MED20 | Mediator of RNA polymerase II transcription subunit 20 | 1.603 | 6708.351 | 5.5488892 | -0.1 |
| Q49AN9 | SNRPG | SNRPG protein | 1.662 | 7101.008 | 6.5366821 | 0.1109375 |
| Q8WUW1 | BRK1 | Protein BRICK1 (BRK1) | 1.858 | 8744.469 | 5.3502808 | -0.546667 |
| R4GN98 | S100A6 | Protein S100-A6 | 1.679 | 9680.649 | 5.2888794 | -0.28 |
| J7HBC9 | RAET1H | Retinoic acid early transcript 1H | 1.936 | 9892.895 | 8.6459351 | -0.397701 |
| C0KRQ8 | CGA | Follicle-stimulating hormone alpha subunit | 1.538 | 10205.24 | 8.3760376 | -0.315217 |
| Q9Y5L4 | TIMM13 | Mitochondrial import inner membrane translocase subunit Tim13 | 1.614 | 10499.5 | 8.4187622 | -0.506316 |
| K7EN20 | HSPBP1 | Hsp70-binding protein 1 | 1.63 | 10613.87 | 7.6793823 | 0.3206186 |
| M0QY80 | ETHE1 | Persulfide dioxygenase ETHE1 | 1.503 | 10631.77 | 7.850769 | -0.206316 |
| E5RHM7 | TTI2 | TELO2-interacting protein 2 | 1.589 | 10989.66 | 4.710022 | -0.512871 |
| D6RHW1 | TAF9 | Transcription initiation factor TFIID subunit 9 | 1.657 | 11569.65 | 6.5571899 | -0.502941 |
| Q9UK45 | LSM7 | U6 snRNA-associated Sm-like protein LSm7 | 1.947 | 11601.76 | 5.1029663 | -0.436893 |
| J3KS95 | FAM96B | Mitotic spindle-associated MMXD complex subunit MIP18 | 1.901 | 11772.87 | 5.6616821 | -0.166981 |
| J3KT60 | C17orf80 | Uncharacterized protein C17orf80 | 1.572 | 12293.87 | 9.6539917 | -0.831193 |
| M0QZH1 | SMG9 | Protein SMG9 | 2.371 | 12439.4 | 9.6878052 | -1.034783 |
| C9JRG3 | DNPEP | Aspartyl aminopeptidase | 1.897 | 13024.2 | 10.750061 | -0.24359 |
| P60866 | RPS20 | 40S ribosomal protein S20 | 1.531 | 13372.01 | 9.9485474 | -0.395798 |
| O75348 | ATP6V1G1 | V-type proton ATPase subunit G 1 | 1.696 | 13756.81 | 8.9258423 | -1.065254 |
| M0QYB4 | CCDC9 | Coiled-coil domain-containing protein 9 | 1.79 | 13812.67 | 10.113953 | -1.257813 |
| K7EQG1 | QPCTL | Glutaminyl-peptide cyclotransferase-like protein | 1.703 | 13979.02 | 11.63446 | -0.112397 |
| Q96A08 | HIST1H2BA | Histone H2B type 1-A | 6.536 | 14166.73 | 10.314514 | -0.586614 |
| O15212 | PFDN6 | Prefoldin subunit 6 | 2.068 | 14581.88 | 8.8285522 | -0.717054 |
| I3L3W0 | ABR | Active breakpoint cluster region-related protein | 1.915 | 14784.99 | 5.6033325 | -0.469173 |
| C9J6N5 | FAM107B | Protein FAM107B | 1.7 | 14843.21 | 8.7523804 | -1.468548 |
| Q9Y2V2 | CARHSP1 | Calcium-regulated heat stable protein 1 | 1.507 | 15891.19 | 8.4054565 | -0.476871 |
| P57105 | SYNJ2BP | Synaptojanin-2-binding protein | 1.682 | 15927.19 | 5.8617554 | -0.168966 |
| K7X1S0 | DIABLO | SMAC-epsilon | 1.934 | 16313.3 | 4.7667847 | -0.695775 |
| P27482 | CALML3 | Calmodulin-like protein 3 | 2.083 | 16889.85 | 4.2979126 | -0.663758 |
| Q9Y3D6 | FIS1 | Mitochondrial fission 1 protein | 1.737 | 16936.78 | 8.8361206 | -0.232895 |
| Q7Z422 | SZRD1 | SUZ domain-containing protein 1 | 1.766 | 16996.13 | 8.9208374 | -1.132895 |
| D6RC14 | MRPL3 | 39S ribosomal protein L3, mitochondrial | 1.586 | 17094.02 | 10.295715 | -0.470323 |
| Q5JXB2 | UBE2NL | Putative ubiquitin-conjugating enzyme E2 N-like | 1.513 | 17376.06 | 5.6592407 | -0.352941 |
| K7EMD6 | SGTA | Small glutamine-rich tetratricopeptide repeat-containing protein alpha | 1.562 | 17790.91 | 4.6746216 | -0.398148 |
| P53816 | HRASLS3 | HRAS-like suppressor 3 | 1.528 | 17935.7 | 7.762146 | -0.169136 |
| B7Z3E2 | PPP6C | Serine/threonine-protein phosphatase 6 catalytic subunit | 1.669 | 18031.87 | 6.1895142 | -0.100633 |
| E5RIA1 | AGPAT6 | Glycerol-3-phosphate acyltransferase 4 | 1.817 | 18195.2 | 8.8651733 | -0.092405 |
| Q9Y3C6 | PPIL1 | Peptidyl-prolyl cis-trans isomerase-like 1 | 1.524 | 18235.84 | 7.77948 | -0.304217 |
| P62487 | POLR2G RPB7 | DNA-directed RNA polymerase II subunit RPB7 | 2.04 | 19293.29 | 5.3252563 | 0.0674419 |
| Q05D40 | CYP51A1 | CYP51A1 protein | 1.544 | 19536.04 | 9.4711304 | 0.3411111 |
| O00762 | UBE2C | Ubiquitin-conjugating enzyme E2 C | 1.613 | 19651.23 | 6.8274536 | -0.393296 |
| Q10589 | BST2 | Bone marrow stromal antigen 2 | 1.566 | 19767.86 | 5.4326782 | 0.0288889 |
| E9PH64 | NDUFB9 | NADH dehydrogenase [ubiquinone] 1 beta subcomplex subunit 9 | 1.804 | 20382.15 | 7.7123413 | -1.110714 |
| I6L9C8 | ZNF428 | Zinc finger protein 428 | 1.583 | 20465.49 | 4.1296997 | -1.355851 |
| K7EQB2 | GLYR1 | Putative oxidoreductase GLYR1 | 1.63 | 20471.62 | 9.5425415 | -0.436757 |
| P18847 | ATF3 | Activating transcription factor 3 | 1.53 | 20574.55 | 8.8025513 | -0.671271 |
| F2X5X4 | HLA-A | MHC class I antigen | 1.618 | 21127.3 | 7.0413208 | -0.904972 |
| P05090 | APOD | Apolipoprotein D | 1.865 | 21274.42 | 5.0582886 | -0.053439 |
| Q8WZA0 | LZIC | Protein LZIC | 1.739 | 21493.39 | 4.8759155 | -0.573684 |
| H7C2Y0 | 41884 | Septin-2 | 1.503 | 21648.94 | 6.0874634 | -0.489894 |
| Q15126 | PMVK PMKI | Phosphomevalonate kinase (PMKase) | 1.917 | 21993.74 | 5.5614624 | -0.43125 |
| Q5QPD0 | NT5DC1 | 5'-nucleotidase domain-containing protein 1 | 1.517 | 22184.32 | 6.1936646 | -0.113333 |
| Q99653 | CHP1 | Calcineurin B homologous protein 1 | 1.611 | 22455 | 4.9805298 | -0.630256 |
| Q6PKD2 | HNRPCL1 | HNRPCL1 protein | 2.059 | 22650.66 | 9.9454956 | -0.631401 |
| P61758 | PFDN3 | Prefoldin subunit 3) | 1.702 | 22656.79 | 6.635437 | -0.713198 |
| H3BRC0 | COQ9 | Ubiquinone biosynthesis protein COQ9 | 2.314 | 22925.32 | 5.5370483 | -0.597101 |
| P10301 | RRAS | Ras-related protein R-Ras (p23) | 1.838 | 23479.19 | 6.4349976 | -0.348165 |
| C9J5C3 | PDCD10 | Programmed cell death protein 10 | 1.902 | 23584.98 | 6.7787476 | -0.504455 |
| P00492 | HPRT1 | Hypoxanthine-guanine phosphoribosyltransferase | 1.752 | 24578.08 | 6.2107544 | -0.136239 |
| A6PWM2 | CRELD2 | Cysteine-rich with EGF-like domain protein 2 | 1.58 | 25128.23 | 5.0137329 | -0.390393 |
| D6RAA6 | TMEM33 | Transmembrane protein 33 | 1.61 | 25222.43 | 9.6217651 | 0.3671171 |
| P28072 | PSMB6 | Proteasome subunit beta type-6 | 1.508 | 25356.33 | 4.7994995 | 0.0343096 |
| Q8TAA5 | GRPEL2 | GrpE protein homolog 2 | 1.517 | 25429.9 | 7.6262817 | -0.4 |
| P82930 | MRPS34 | 28S ribosomal protein S34 | 1.618 | 25649.14 | 9.982605 | -0.767431 |
| Q6P5S8 | IGK@ | IGK@ protein | 2.755 | 25771.45 | 5.9429321 | -0.288559 |
| Q07817 | BCL2L1 | Bcl-2-like protein 1 | 1.549 | 26047.58 | 4.8584595 | -0.345064 |
| Q96CF2 | CHMP4C | Charged multivesicular body protein 4c | 1.535 | 26409.29 | 5.8328247 | -0.924034 |
| Q9UKD2 | MRTO4 | mRNA turnover protein 4 homolog | 1.614 | 27559.03 | 8.3366089 | -0.712134 |
| Q8N129 | CNPY4 | Protein canopy homolog 4 | 2.904 | 28308.24 | 4.5964966 | -0.696774 |
| E9PK01 | EEF1D | Elongation factor 1-delta | 1.569 | 28819.67 | 4.9285278 | -0.621073 |
| E9PMD7 | PPP1CA | Serine/threonine-protein phosphatase | 1.741 | 28896.37 | 4.7373657 | -0.252964 |
| A6NCD4 | C10orf131 | Uncharacterized protein C10orf131 | 1.852 | 29613.33 | 5.9711304 | -0.767969 |
| E7EPA1 | PRPSAP2 | Phosphoribosyl pyrophosphate synthase-associated protein 2 | 1.537 | 29640.32 | 9.0841675 | 0.0380597 |
| P29218 | IMPA1 | Inositol monophosphatase 1 | 1.797 | 30187.2 | 5.1599731 | 0.0433213 |
| Q9Y3B9 | RRP15 | RRP15-like protein | 1.74 | 31482.54 | 5.3895874 | -1.114184 |
| O00165 | HAX1 HS1BP1 | HCLS1-associated protein X-1 | 1.59 | 31618.81 | 4.7599487 | -0.969534 |
| K7ENQ8 | CYTH1 | Cytohesin-1 | 2.369 | 31649.22 | 4.8965454 | -0.628309 |
| O00625 | PIR | Pirin | 1.653 | 32111.62 | 6.4240112 | -0.562069 |
| Q49AC9 | PTPRM | PTPRM protein | 1.823 | 32278.27 | 6.2449341 | -0.376344 |
| Q9BRL6 | SRSF8 | Serine/arginine-rich splicing factor 8 | 1.649 | 32285.83 | 11.716003 | -1.581915 |
| B4DM41 | RFC4 | Replication factor C (Activator 1) 4) | 1.941 | 33673.37 | 9.6088257 | -0.155776 |
| Q15785 | TOMM34 | Mitochondrial import receptor subunit TOM34 | 1.761 | 34557.54 | 9.119812 | -0.609385 |
| B7ZAB3 | B3GAT3 | Galactosylgalactosylxylosylprotein 3-beta-glucuronosyltransferase 3 | 1.634 | 34623 | 7.71698 | -0.172698 |
| Q9HC38 | GLOD4 | Glyoxalase domain-containing protein 4 | 1.754 | 34791.6 | 5.3981323 | -0.383067 |
| F2Z393 | TALDO1 | Transaldolase | 2.286 | 35326.93 | 9.0747681 | -0.181447 |
| P55735 | SEC13 | Protein SEC13 homolog | 1.786 | 35538.6 | 5.2201538 | -0.371739 |
| H0YK42 | SNX1 | Sorting nexin-1 | 1.51 | 35590.82 | 6.3092651 | -0.619737 |
| O60587 | FTF | Alpha1-fetoprotein transcription factor short variant | 1.58 | 36408.08 | 9.0170288 | -0.443344 |
| Q8TB03 | CXorf38 | Uncharacterized protein CXorf38 | 1.526 | 36668.07 | 5.9207153 | -0.495298 |
| O95400 | CD2BP2 | CD2 antigen cytoplasmic tail-binding protein 2 | 1.627 | 37644.42 | 4.494812 | -0.828152 |
| B4DT77 | ANXA7 | Annexin | 1.529 | 37802.99 | 6.7711792 | -0.41875 |
| Q8N0V3 | RBFA C18orf22 | Putative ribosome-binding factor A, | 2.716 | 38357.34 | 8.006897 | -0.648105 |
| A6NED2 | RCCD1 | RCC1 domain-containing protein 1 | 1.607 | 40076.69 | 5.1828003 | -0.25133 |
| Q9BUA3 | C11orf84 | Uncharacterized protein C11orf84 | 1.695 | 41034.54 | 4.9175415 | -0.769816 |
| Q8N8C0 | ZNF781 | Zinc finger protein 781 | 2.49 | 41524.14 | 10.395081 | -0.06169 |
| Q9Y570 | PPME1 | Protein phosphatase methylesterase 1 | 1.679 | 42313.16 | 5.6691284 | -0.289119 |
| Q9NYB0 | TERF2IP | Telomeric repeat-binding factor 2-interacting protein 1 | 1.787 | 44257.58 | 4.6377563 | -0.763409 |
| Q13641 | TPBG | Trophoblast glycoprotein | 1.527 | 46029.36 | 6.3544312 | 0.0111905 |
| A6NGH8 | ANKRD61 | Ankyrin repeat domain-containing protein 61 | 1.75 | 46138.05 | 9.1036987 | -0.064833 |
| Q9BQ95 | ECSIT | Evolutionarily conserved signaling intermediate in Toll pathway | 1.745 | 49145.52 | 5.8927612 | -0.446404 |
| Q3ZCM7 | TUBB8 | Tubulin beta-8 chain | 1.936 | 49773.4 | 4.7861938 | -0.35518 |
| Q9Y6M4 | CSNK1G3 | Casein kinase I isoform gamma-3 | 1.556 | 51385.83 | 9.2909546 | -0.747651 |
| P63151 | PPP2R2A | Serine/threonine-protein phosphatase 2A 55 kDa regulatory subunit B alpha isoform | 1.534 | 51689.39 | 5.819397 | -0.610515 |
| Q9P109 | GCNT4 | Beta-1,3-galactosyl-O-glycosyl-glycoprotein beta-1,6-N-acetylglucosaminyltransferase 4 | 1.61 | 53049.45 | 8.4824829 | -0.22936 |
| Q99640 | PKMYT1 MYT1 | Membrane-associated tyrosine- and threonine-specific cdc2-inhibitory kinase | 2.853 | 54518.4 | 6.4766235 | -0.281764 |
| Q96SQ9 | CYP2S1 | Cytochrome P450 2S1 | 2.153 | 55813.62 | 8.796814 | -0.024405 |
| Q9H9C1 | 3 SPE39 | Spermatogenesis-defective protein 39 homolog | 2.107 | 57002.21 | 6.9414673 | -0.647667 |
| Q9Y6R9 | CCDC61 | Coiled-coil domain-containing protein 61 | 2.258 | 57365.22 | 10.273132 | -0.926367 |
| P05187 | ALPP | Alkaline phosphatase, placental type | 1.62 | 57950.65 | 5.864563 | -0.207477 |
| Q96LT9 | RBM40 | RNA-binding protein 40 | 1.905 | 58572.01 | 7.6089478 | -0.718762 |
| Q9NUL7 | DDX28 | Probable ATP-dependent RNA helicase DDX28) | 1.622 | 59577.93 | 10.427063 | -0.122778 |
| Q96PX6 | CCDC85A | Coiled-coil domain-containing protein 85A | 1.804 | 59972.29 | 9.0308228 | -0.962387 |
| Q9P270 | SLAIN2 | SLAIN motif-containing protein 2 | 1.552 | 62539.97 | 9.4832153 | -0.824957 |
| Q13131 | PRKAA1 AMPK1 | 5'-AMP-activated protein kinase catalytic subunit alpha-1 | 1.588 | 64005.87 | 8.3223267 | -0.461002 |
| F8VQE1 | LIMA1 | LIM domain and actin-binding protein 1 | 1.692 | 66987.63 | 5.6256714 | -1.049498 |
| Q16822 | PCK2 | Phosphoenolpyruvate carboxykinase) | 1.649 | 70726.18 | 7.5687866 | -0.278438 |
| Q9NPI1 | BRD7 | Bromodomain-containing protein 7 | 1.532 | 74134.66 | 5.9995728 | -0.94086 |
| Q9NYY8 | FASTKD2 | FAST kinase domain-containing protein 2 | 1.545 | 81458.65 | 8.3067017 | -0.048028 |
| Q9BRZ2 | TRIM56 | E3 ubiquitin-protein ligase TRIM56 | 1.635 | 81483.26 | 8.0216675 | -0.36543 |
| Q7KZI7 | MARK2 | Serine/threonine-protein kinase MARK2 | 1.548 | 87906.04 | 9.7272339 | -0.678426 |
| Q86XL3 | ANKLE2 | Ankyrin repeat and LEM domain-containing protein 2 | 1.9 | 104108.7 | 6.6574097 | -0.61162 |
| Q9P253 | VPS18 | Vacuolar protein sorting-associated protein 18 homolog (hVPS18) | 1.667 | 110179.8 | 5.7202759 | -0.268756 |
| P52333 | JAK3 | Tyrosine-protein kinase JAK3 | 3.103 | 125092.2 | 6.769104 | -0.147598 |
| B7ZLC9 | GEMIN5 | GEMIN5 protein | 1.838 | 168423.2 | 6.1713257 | -0.396417 |
| Q562E7 | WDR81 | WD repeat-containing protein 81 | 1.617 | 211685.1 | 5.3706665 | -0.250283 |
| Q14669 | TRIP12 | E3 ubiquitin-protein ligase TRIP12 | 1.657 | 220421.7 | 8.7572632 | -0.50758 |
| Q9P2D3 | HEATR5B | HEAT repeat-containing protein 5B | 1.638 | 224290.2 | 6.7672729 | 0.0954128 |
